# Robust Covalent Aptamer Strategy Enables Sensitive Detection and Enhanced Inhibition of SARS-CoV-2 Proteins

**DOI:** 10.1021/acscentsci.2c01263

**Published:** 2023-01-02

**Authors:** Dan Wang, Jing Zhang, Zhiyong huang, Yuhang Yang, Ting Fu, Yu Yang, Yifan Lyu, Jianhui Jiang, Liping Qiu, Zehui Cao, Xiaobing Zhang, Qimin You, Yuankui Lin, Zilong Zhao, Weihong Tan

**Affiliations:** † Molecular Science and Biomedicine Laboratory (MBL), State Key Laboratory of Chemo/Biosensing and Chemometrics, College of Chemistry and Chemical Engineering, Aptamer Engineering Center of Hunan Province, Hunan University, Changsha, Hunan 410082, China; ‡ Zhejiang Cancer Hospital, Hangzhou Institute of Medicine (HIM), Chinese Academy of Sciences, Hangzhou, Zhejiang 310022, China; § Institute of Molecular Medicine (IMM), Renji Hospital, Shanghai Jiao Tong University School of Medicine and College of Chemistry and Chemical Engineering, Shanghai Jiao Tong University, Shanghai 200240, China; ∥ LIMES Chemical Biology Unit, Universität Bonn, 53121 Bonn, Germany; ⊥ Ustar Biotechnologies (Hangzhou) Ltd., Hangzhou, Zhejiang 310053, China

## Abstract

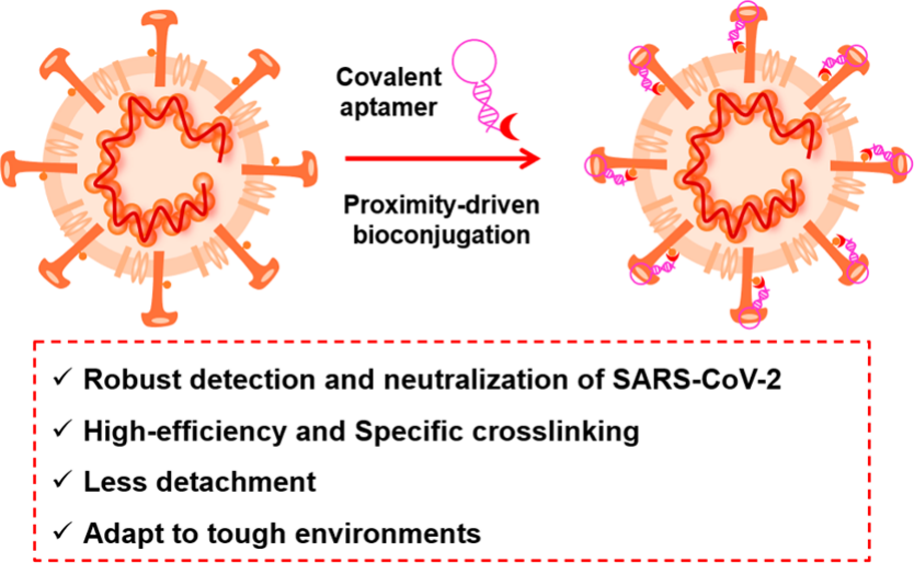

Aptamer-based detection and therapy have made substantial progress with cost control and easy modification. However, the conformation lability of an aptamer typically causes the dissociation of aptamer–target complexes during harsh washes and other environmental stresses, resulting in only moderate detection sensitivity and a decreasing therapeutic effect. Herein, we report a robust covalent aptamer strategy to sensitively detect nucleocapsid protein and potently neutralize spike protein receptor binding domain (RBD), two of the most important proteins of SARS-CoV-2, after testing different cross-link electrophilic groups via integrating the specificity and efficiency. Covalent aptamers can specifically convert aptamer–protein complexes from the dynamic equilibrium state to stable and irreversible covalent complexes even in harsh environments. Covalent aptamer-based ELISA detection of nucleocapsid protein can surpass the gold standard, antibody-based sandwich ELISA. Further, covalent aptamer performs enhanced functional inhibition to RBD protein even in a blood vessel-mimicking flowing circulation system. The robust covalent aptamer-based strategy is expected to inspire more applications in accurate molecular modification, disease biomarker discovery, and other theranostic fields.

## Abstract 2

By a proximity-driven bioconjugation strategy, we herein develop robust covalent aptamer probes to potently detect and block nucleocapsid protein and RBD protein of SARS-CoV-2, respectively.

## Introduction

Aptamers, also termed as chemical antibodies, are single-stranded DNA/RNA sequences generated by applying an in vitro-directed evolution technique named as systematic evolution of ligands by exponential enrichment (SELEX) against targets of interest.^1−4^ Aptamers recognize their cognate targets by folding into particular conformations. Based on the low cost, easy chemical synthesis, programmability, and compatibility to various amplification methods and high-throughput techniques, aptamers have been extensively utilized in various applications including biological detection and targeted therapeutics.^5−8^ The performance of aptamers in biological detection and targeted therapy are mainly determined by their binding avidity and specificity. However, the conformation of aptamers is labile based on their flexible backbone and dynamic hydrogen bond between base pairs, and easily susceptible to environmental conditions (e.g., multiple washing), undermining the binding avidity of aptamer and the stability of aptamer–target complexes.^9−12^ Therefore, aptamers usually present moderate performances in detection and therapy compared to commercial antibodies. To improve the binding avidity and reduce the dissociation of aptamer–target complexes, the molecular assembly strategy, which generates bivalent or multiple aptamers, and the molecular engineering strategy, which integrates hydrophobic groups in an aptamer to increase the binding mode, e.g., hydrophobic interaction, have been developed.^13−18^ However, these strategies do not change the noncovalent binding pattern between aptamer and target, and only moderately improve the binding avidity of aptamer and the stability of the aptamer–target complexes.

**Figure 1:**
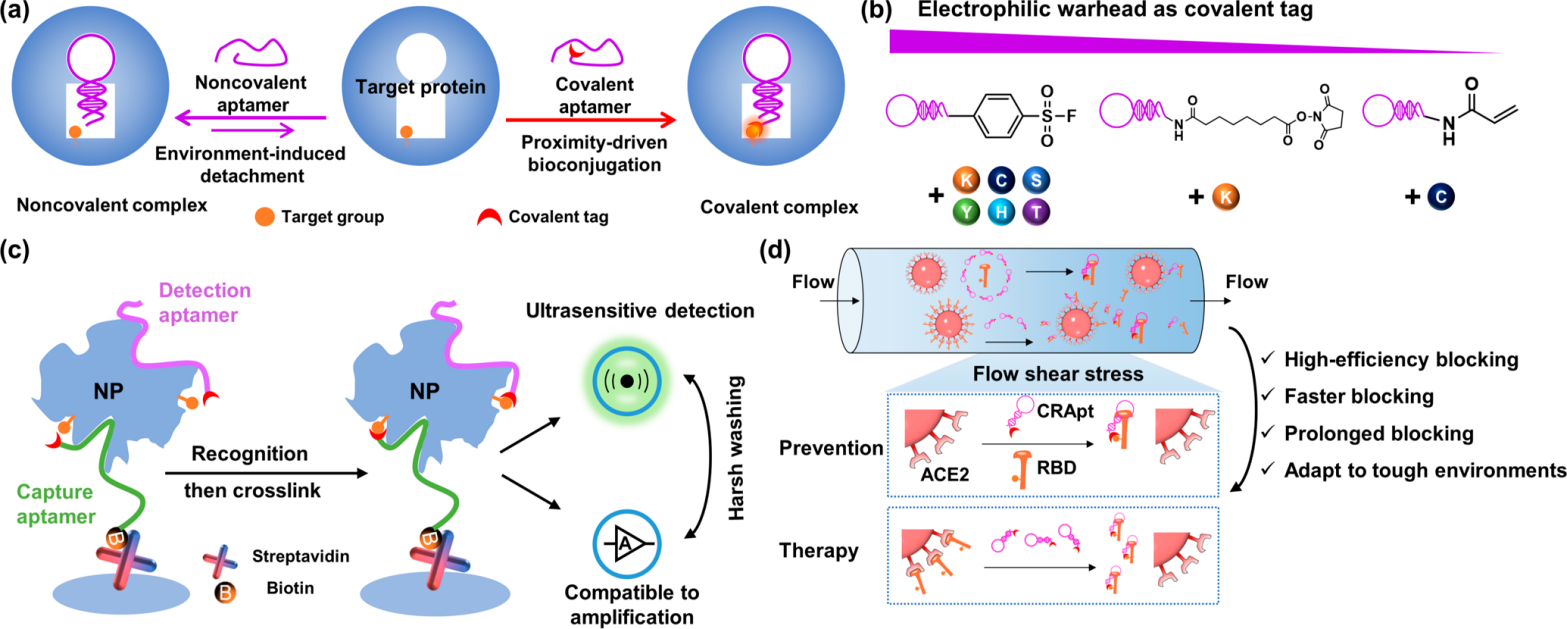
Covalent aptamer-based strategies for detection and functional blocking of target protein by specific proximity-mediated cross-link. (a) Schematic illustration of environmental stress-resistant covalent aptamer for recognition of target protein compared to conventional noncovalent aptamer. (b) Comparison of reactive affinities and targeted amino acids of three covalent tags. (c) Schematic illustration of the covalent aptamer-based sandwich ELISA with or without signal amplification strategy for ultrasensitive detection of NP of SARS-CoV-2 under harsh washing. (d) Schematic illustration of the covalent aptamer-mediated neutralization of SARS-CoV-2.

Inspired by the on-the-shelf covalent small-molecule drugs and hottest covalent proteins,^19−24^ recently several electrophilic groups have been considered as warheads to modify aptamers to develop covalent aptamers. For example, Tivon et al. have developed tosyl or sulfonamide-modified covalent aptamers to specifically cross-link and label proteins of interest (POI).^12^ In addition, sulfur(VI) fluoride exchange (SuFEx) group-modified covalent aptamers have been developed to enhance the blocking thrombin activity and SARS-CoV-2 RBD-ACE2 interaction.^25,26^ Covalent aptamers can convert conventional aptamer–target noncovalent complexes into covalent complexes, thus preventing probe detachment and standing up to various environmental stresses (Figure 1a).

However, there are still several limitations in the field of emerging covalent aptamers. First, few studies are performed to systematically investigate the cross-link efficiency, cross-link specificity and reaction kinetics of covalent aptamers composed of the same aptamer and entirely different covalent warheads, on the same POI. Molecular recognition-mediated covalent cross-link may cause nonspecific reaction with nontargeted molecules.^27^ Therefore, it is important for covalent ligands, including covalent kinase inhibitors, covalent proteins and emerging covalent aptamers, to elucidate the balance issue between cross-link efficiency and cross-link specificity.^28−30^ Second, there is still a lack of systematic and direct comparisons among the covalent aptamers, noncovalent aptamers, and commercial antibodies.^12,25,26^ The direct comparisons in the same conditions will be beneficial to demonstrate the performance of a covalent aptamer. Finally, currently the concept and the application of covalent aptamers are still in infancy. Besides the three examples mentioned previously, the most common reports use diazirine derivatives as the photoaffinity labels to conjugate aptamers or DNAs with POI for cross-link modification in a site-specific way.^29,31−35^ Therefore, it is important to promote the concept development and expand the application fields of the covalent aptamers.

To address the above issues, we herein conjugate three nominated electrophilic warheads with aptamers to develop covalent aptamers, functionalized as detecting probes and functional blocking neutralizers of SARS-CoV-2 proteins (Figure 1). Sulfuryl fluoride-modified aptamers (SF-Apt) can covalently cross-link with proximal nucleophilic groups of lysine (K), cysteine (C), serine (S), tyrosine (Y), histidine (H), and threonine (T) of POI.^25^ NHS (*N*-hydroxysuccinimide)-modified aptamers (NHS-Apt) can cross-link with ε-amino residues of lysine of POI. Acrylamide-modified aptamers (Acr-Apt) can cross-link with mercapto group of cysteine in POI (Figure 1b).^36,37^ After reasonable selection of covalent tags, it is expected that POI bound aptamer can expose its covalent tag hiding behind flexible strands to facilitate proximity-driven “click” bioconjugation with target groups to resist various environmental interferences (Figure 1a), making this platform promising in various analytical methods and therapeutic applications.

As a proof-in-concept, we choose corresponding covalent aptamers to detect nucleocapsid protein (NP) and neutralize spike protein receptor binding domain (RBD) of SARS-CoV-2. NP, the most conserved and abundant structural protein of SARS-CoV-2, is the most optimal choice for early detection and reliable diagnosis.^38,39^ RBD, the crucial protein of SARS-CoV-2 to recognize and infect ACE2 protein of host cell, is the neutralization target to inhibit viral infection. Therefore, after recognizing NP and RBD, these covalent aptamers can be expected to bring the covalent tags proximity to NP and RBD, and facilitate the formation of covalent aptamer–targeted proteins complexes (Figure 1a). Based on the systematically investigation on the cross-link efficacy, specificity, and kinetics of the three covalent tags, suitable covalent aptamers were selected for application studies compared to commercial antibodies and noncovalent aptamers. On the one hand, two covalent NP-targeted aptamers were functionalized as capture probe and detection probe, respectively, to execute sandwich ELISA (enzyme-linked immunosorbent assay) for specially and ultrasensitively detecting NP (Figure 1c). In addition, covalent aptamer can easily integrate various amplification methods under harsh washing (Figure 1c). On the other hand, covalent RBD-targeted aptamer demonstrates better neutralization (e.g., prevention or therapy) ability compared to classic antibodies and traditional aptamers, including higher blocking efficiency, faster blocking, prolonged blocking, and stronger adaption to tough environment (e.g., flow shear stress, Figure 1d). Our results reveal that the NHS-based covalent aptamers display excellent binding avidity and form stable aptamer–targeted protein complexes. Therefore, the covalent aptamer-based sandwich ELISA and RBD neutralizer demonstrate more excellent performance than noncovalent aptamer/commercial antibody-based ELISA and neutralizer. Taken together, our study not only expands the application field of the covalent aptamers but also sets a new stage to promote the development of aptamer-based detection techniques and therapeutics. Covalent strategy can revolutionize the applications of aptamers in diagnosis, molecular imaging, disease therapy, and other biomedical applications by converting noncovalent binding pattern into covalent binding pattern between aptamer and targeted proteins.

## Results and Discussion

### Synthesis and Cross-Link Characterization of Three Nominated Covalent Aptamers

**Figure 2:**
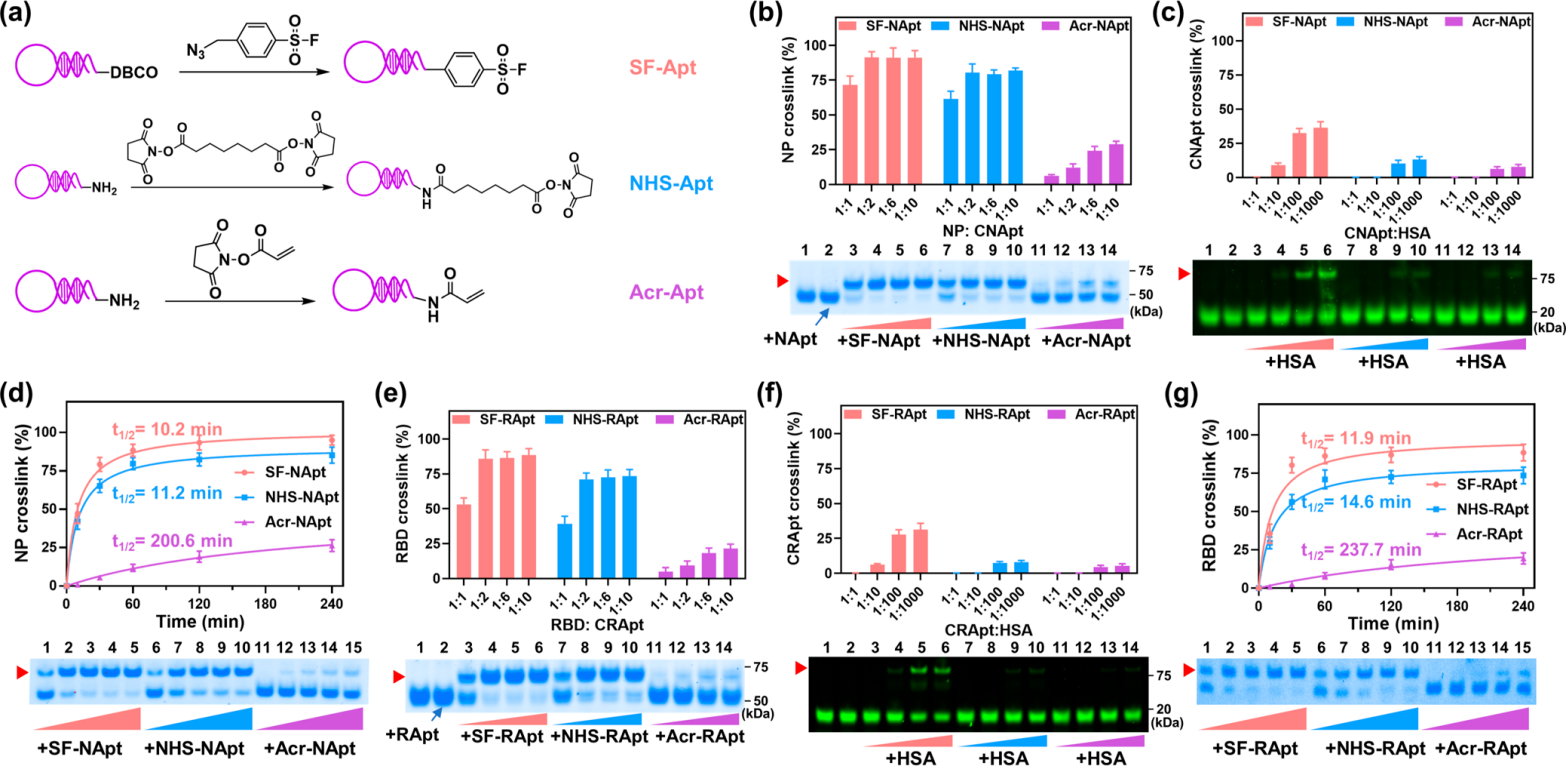
Synthesis and characterization of three nominated covalent aptamers. (a) Brief synthetic scheme of three covalent aptamers. (b) Reducing SDS-PAGE analysis of cross-link efficiency after 400 nM NP reacted with different concentrations of CNApt for 4 h (protein stained by Coomassie Blue). Lane 1: NP; lane 2: NP + noncovalent NApt; lanes 3–14: NP + different equivalents of CNApt. (c) Reducing SDS-PAGE analysis of cross-link efficiency after 400 nM FAM-labeled CNApt reacted with different concentrations of HSA (gel presented with FAM field). Lane 1: FAM-labeled noncovalent NApt; lane 2: lane 1 + HSA; lanes 3–14: FAM-labeled CNApt + different equivalents of HSA. (d) Reducing SDS-PAGE analysis of cross-link kinetics between 400 nM NP and 6 eq. CNApt (protein stained by Coomassie Blue). (e) Reducing SDS-PAGE analysis of cross-link efficiency after 400 nM RBD reacted with different concentrations of CRApt (protein stained by Coomassie Blue). Lane 1: RBD; lane 2: RBD + noncovalent RApt; lanes 3–14: RBD + different equivalents of CRApt. (f) Reducing SDS-PAGE analysis of cross-link efficiency after 400 nM FAM-labeled CRApt reacted with different concentrations of HSA for 4 h (gel presented with FAM field). Lane 1: FAM-labeled noncovalent RApt; lane 2: lane 1 + HSA; lanes 3–14: FAM-labeled CRApt + different equivalents of HSA. (g) Reducing SDS-PAGE analysis of cross-link kinetics between 400 nM RBD and 6 eq. CRApt (protein stained by Coomassie Blue). All data are mean ± s.d. from at least two replicates.

To reduce the impact of the covalent tags on aptamer’s affinity and decrease the spatial hindrance of aptamer on the movement of covalent tags, 4-(azidomethyl) benzenesulfonyl fluoride (Figures S1–S4), disuccinimidyl suberate and 2,5-dioxopyrrolidin-1-yl acrylate were attached to 5′-termina of NP-targeted aptamers (Apt61, Apt15, Apt48, and Apt58, Table S1)^40^ or RBD-targeted aptamer (RApt, Table S1) to prepare covalent aptamers via strain-promoted azide–dibenzocyclooctyne cycloaddition or NHS-mediated conjugation (Figure 2a). In this study, 16 covalent aptamers were prepared and further determined by ESI-MS (Table S2).

To systematically investigate the performance of covalent aptamers with different covalent tags, three NP-targeted covalent aptamers (Apt61), SF-NApt, NHS-NApt and Acr-NApt, were chosen for cross-link analysis. The covalent cross-link efficiency of the three CNApts was first evaluated by using the reducing SDS-PAGE (sodium dodecyl sulfate-polyacrylamide gel electrophoresis) to analyze the formation of the covalent aptamer–target complexes after 400 nM NP was incubated with covalent aptamers at different concentration ranging from 400 nM to 4 μM for 4 h in PBS buffer (pH 7.4). The reducing SDS-PAGE could damage the noncovalent interaction between aptamer and protein (lane 2 in Figure 2b).^28,41^ Therefore, the upper bands represented the covalent cross-link aptamer–protein complexes, and the covalent cross-link efficiency could be determined by analyzing the intensity ratio between the upper band and the lower band. SF-NApt and NHS-NApt could result in the conversion of ∼91% and ∼80% of NP into covalent cross-link complexes, respectively, when the ratio of NP to covalent aptamer was 1:2. However, Acr-NApt only caused the conversion of ∼12% of NP into covalent aptamer–target complexes at the same conditions (Figure 2b). The results revealed that the covalent tags SF and NHS could react more efficiently with targeted proteins than Acr tag after the covalent aptamers recognized targeted proteins. Interestingly, Figure 2b revealed that the stoichiometric ratio of NApt and NP was 1:1, as evidenced by the fact that the molecule weight of the cross-link product was not changed even when the ratio of NP to CNApt was increased to 1:10.

Next, the cross-link specificity of the three covalent aptamers was investigated by employing various concentrations of human serum albumin (HSA) to incubate with a constant 400 nM 6-Carboxyfluorescein (FAM)-labeled CNApt for 4 h in PBS buffer. Figure 2c revealed that SF-NApt displayed the highest unwanted cross-link efficiency on nontargeted protein HSA than NHS-NApt and Acr-NApt. For example, FAM-labeled SF-NApt could cause the conversion of ∼9% HSA into the cross-link complex when the ratio of the covalent aptamer and HSA was 1:10. Conversely, FAM-labeled NHS-NApt and FAM-labeled Acr-NApt did not cause the formation of covalent aptamer-HSA cross-link at the same conditions. The high nonspecific cross-link efficiency of SF was probably that it was more active than other two tags and tended to cross-link with nontargeted protein during the random collision.

Finally, the reaction kinetics of the three covalent aptamers and the targeted protein were evaluated. The half-life of a reaction (*t*_1/2_), which is defined as the amount of time needed for the aptamer-target cross-link to increase to the half of the maximum cross-link, was used to evaluate the reaction kinetics of the three covalent aptamers and NP. As shown in Figure 2d, *t*_1/2_ of SF-NApt, NHS-NApt ,and Acr-NApt with NP was 10.2, 11.2, and 200.6 min, respectively, when the ratio of NP and covalent aptamer was 1:6. When the ratio of NP and covalent aptamer was changed to 1:2, *t*_1/2_ of SF-NApt, NHS-NApt, and Acr-NApt with NP was 12.7, 22.8, and >240 min, respectively (Figure S5a). The results displayed that SF-NApt and NHS-NApt had good reaction kinetics, which could facilitate the application of the covalent aptamers.

We also prepared three RBD-targeted covalent aptamers (CRApts), SF-RApt, NHS-RApt, and Acr-RApt, by conjugating electrophilic tags with the RBD-targeted aptamer (Table S1,S2).^42^ Then, the cross-link efficiency, the cross-link specificity and the reaction kinetics of the three CRApts with RBD protein were investigated by the reducing SDS-PAGE. SF-RApt, NHS-RApt, and Acr-RApt cause the conversion of >85%, > 70%, and <20% of RBD protein into the aptamer–RBD complexes, respectively, after incubation for 4 h (Figure 2e). As for the selectivity of cross-link, NHS-RApt and Acr-RApt showed much lower nonspecific cross-link with HSA than SF-RApt at all ratios of the covalent aptamers to HSA. SF-RApt caused ∼30% unwanted cross-link with HSA at saturated ratio of 1:100 while NHS-RApt and Acr-RApt showed only ∼8% and ∼5% cross-link with HSA respectively at saturated ratio of 1:100 (Figure 2f). In addition, NHS-RApt also exhibited good reaction kinetics as SF-RApt: both got a *t*_1/2_ of 10–20 min (Figure 2g, Figure S5b). Taken together, the NHS tag was chosen to develop a covalent aptamer for further study.

### Binding Affinity and Environmental-Stress-Resistant Ability of Covalent NP Aptamer

**Figure 3:**
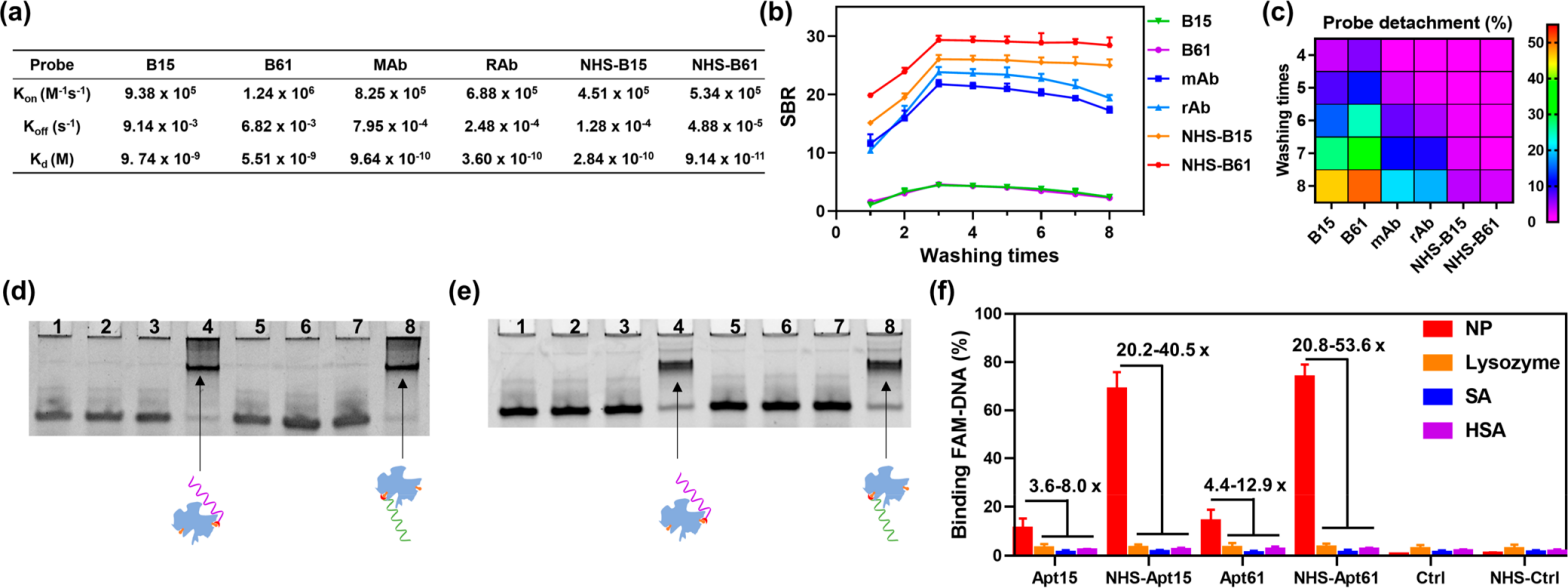
Covalent aptamer strategy enhanced the antienvironment stress capacities of NApt for higher specific binding ability. (a) *K*_d_ measurements of different NP probes by SPR. (b) SBR analysis after multiple washing times in direct ELISA assays using different probes. (c) Heat-map analysis of probe detachment from NP after washing three times (0% detachment) based on (b). (d–e) Gel electrophoresis analysis of noncovalent aptamer–protein complexes and covalent aptamer–protein complexes treated with (d) excess EDTA (20 mM) and (e) 7 M urea. Lane 1: B61, lane 2: B61 + NP + EDTA or urea, lane 3: NHS-B61, lane 4: NHS-B61 + NP + EDTA or urea, lane 5: B15, lane 6: B15 + NP + EDTA or urea, lane 7: NHS-B15, lane 8: NHS-B15 + NP + EDTA or urea. (f) Binding specificity test of 20 nM FAM-DNA incubated with 10 μg/mL NP, lysozyme, SA and 1 mg/mL HSA precoated on SA-free ELISA wells. All data are mean ± s.d., *n* = 2.

ELISA is the gold standard for sensitive and high-throughput immunoassays for targets of interest, and undergoes multiple washing, which is suitable to verify the enhanced detection ability of covalent aptamers. To obtain the best aptamer pair for ELISA assay, four pairs of NP aptamers, Apt15/Apt48 paired with Apt58/Apt61,^40^ were tested by aptamer-based sandwich ELISA. The Apt15-Apt61 aptamer pair showed the highest signal-background-ratio (SBR, the signal ratio between tested samples and blank samples). Therefore, biotin-labeled Apt15 and Apt61 (B15, B61) were used as capture probe and detection probe, respectively (Figure S6). Meanwhile, to systematically evaluate the performance of the covalent aptamer-based ELISA, a noncovalent aptamer-based ELISA and a commercially available two antibodies-based (mAb, rAb) sandwich ELISA Kit for NP (NAb ELISA) were used as controls. Before performing ELISA, the binding abilities of noncovalent aptamers (B15, B61), commercial antibodies (mAb, rAb), and covalent aptamers (NHS-B15, NHS-B61) were investigated by surface plasmon resonance (SPR) assay. There were ignorable differences in the association rate constants (*K*_on_) between aptamers and antibodies. However, the dissociation rate constants (*K*_off_) of aptamers were ∼10-fold higher those of antibodies, which might be caused by the conformation lability of aptamers. Therefore, the noncovalent aptamer presented a 10-fold lower binding affinity (*K*_d_) to NP than antibodies (Figure 3a, Figure S7). The introduction of the NHS tag had a marginal effect on *K*_on_, but substantially decreased *K*_off_ of both aptamers via forming covalent aptamer–target protein complex, thereby resulting in better *K*_d_ (Figure 3a, FigureS7). It is worth noting that *K*_off_ is not zero because the covalent cross-link efficiency is not 100% in dynamic analysis assay. Our results clearly displayed that the binding affinity of the covalent aptamers were 30–60 times higher those of noncovalent aptamers and could be comparable with, or even better than those of, antibodies tested in this study.

To highlight the merits of the covalent aptamer–protein complexes, the abilities of the covalent aptamers to resist various environmental pressures, such as multiple washings, EDTA or urea, which impaired aptamer–target complexes, were tested. We performed direct ELISA assays (i.e, coating NP first and then incubating tested probes) to analyze SBR to test the ability of covalent aptamers, noncovalent aptamers and antibodies to resist multiple washing (Figure 3b). After washing 8 times, the detachment proportions were ∼20% and >45% for antibody–NP complexes and noncovalent aptamer–NP complexes respectively; however, the dissociation ratio of covalent aptamer–NP complexes was less than 5% (Figure 3c, Figure S8). In addition, the covalent aptamer–protein complexes could stand up to EDTA, which can chelate Mg^2+^ and destroy the conformation of aptamer, and urea, which can impair hydrogen bond (Figure 3d,e). Therefore, the covalent aptamer-based detection strategy could be anticipated to achieve high specificity by washing with pure water (or EDTA solution) and urea solution to eliminate nonspecific absorption, promoting the huge detection potential in severe environments, including low salt milieu and urine.

After proving the antiwashing robustness, we next evaluated the specificity of proximity-driven covalent cross-link. First, the interaction of NHS-Ctrl (control DNA) and NP was analyzed by gel electrophoresis. NHS-Ctrl could not bind to or cross-link with NP, which demonstrated that the cross-link between NHS tag and NP is mediated by the specific recognition of aptamer (Figure S9). Then, the effect of NHS tag on aptamer specificity was further investigated by measuring the binding of FAM-labeled covalent aptamers, noncovalent aptamers, and control DNAs on NP, lysozyme, streptavidin (SA), and BSA by direct ELISA assays. The 5- to 6-fold binding of covalent aptamers against noncovalent aptamers contributed to several tens-fold selectivity between NP and nontargeted proteins (Figure 3f, Figure S10). The specificity of NHS-aptamers was also further confirmed when the NHS-BCtrl had only negligible binding to target NP and other proteins (Figure 3f, Figure S10). These results were attributed to the covalent and specific cross-link of CNApt between NHS tag and its proximal NP lysine residues. Then we studied the detailed cross-link sites by LC-MS/MS and molecular docking simulation. LC-MS/MS analysis showed that K-338, K-299 and K-355 could not been recognized and digested by trypsin because they had been modified by NHS-Apt15 and NHS-Apt61. Molecular docking simulation further provided the structural information for the cross-link that the distance between the nitrogen atom in primary amino group of lysine residue (K-338) in NP and the carbon atom in the carbonyl group next to NHS group in NHS-Apt15 was 2.7 Å, and the distance between the nitrogen atom in primary amino group of lysine residues (K-299 and K-335) and the carbon atom in the carbonyl group next to NHS group in NHS-Apt61 was 4.3 and 1.8 Å, respectively (Figure S11).

### Covalent Aptamer-Based Sandwich ELISA Detection of NP

**Figure 4:**
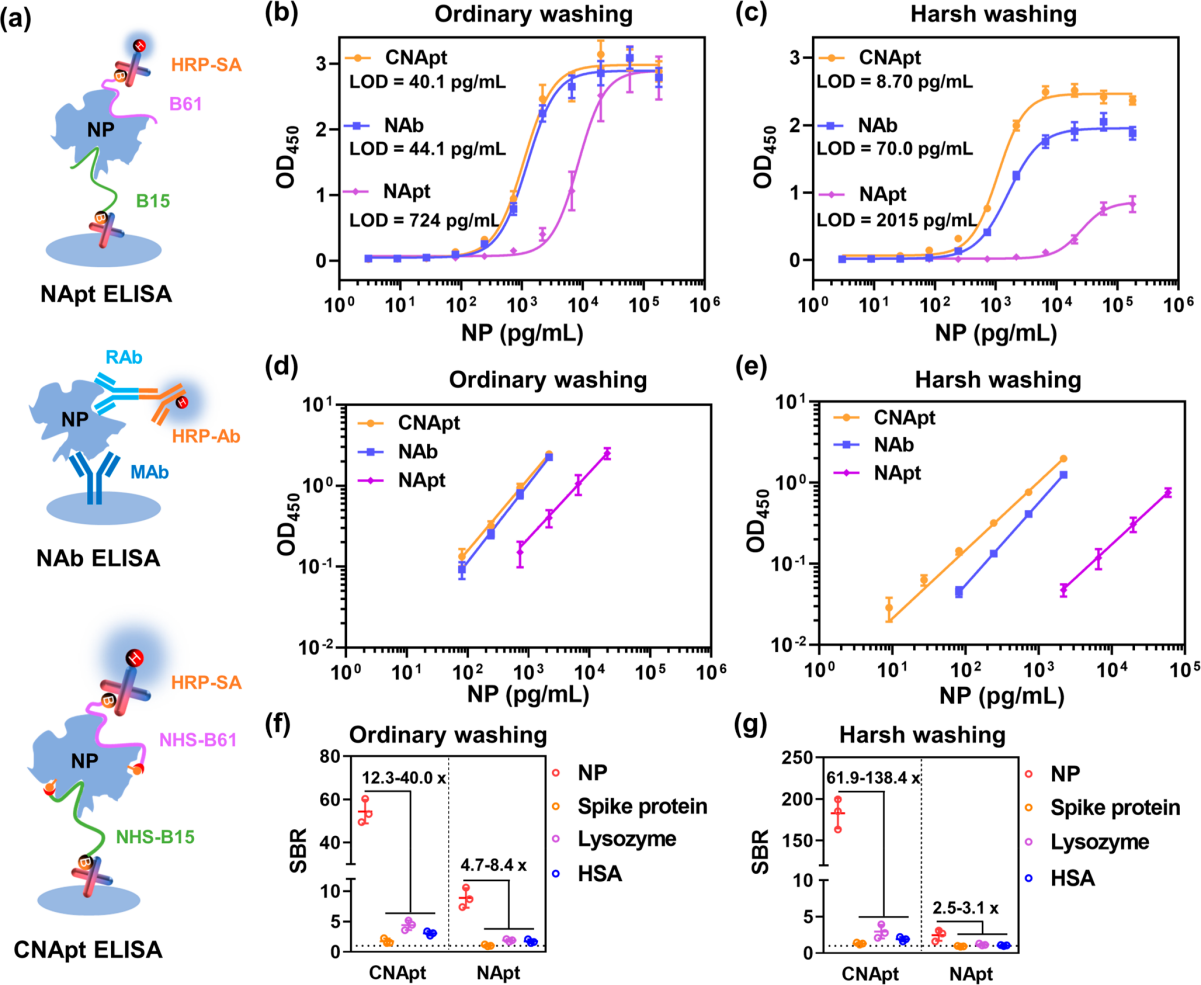
ELISA detection of NP by using various probes under different washing buffer. (a) Brief schematic illustrations of NApt ELISA, NAb ELISA and CNApt ELISA. (b) Sensitivity test of NP dissolving in 10% FBS under ordinary washing. (c) Sensitivity test of NP dissolving in 10% FBS under harsh washing. (d) Linear range results under ordinary washing. (e) Linear range results under harsh washing. (f) Specificity test of CNApt ELISA and NApt ELISA under ordinary washing. (g) Specificity test of CNApt ELISA and NApt ELISA under harsh washing. NP, SP, and lysozyme were 1 ng/mL, HSA was 100 ng/mL. All data are mean ± s.d., *n* = 3.

Because NP (isoelectric point, 10.07) is positively charged in the detection PBS buffer (pH 7.4), to enhance the specificity of binding and detection, salmon sperm DNA, biotin-labeled library DNA (BLib) and high ionic strength were optimized to shield the electrostatic interaction between positively charged NP and negatively charged aptamer, and block nonspecific binding absorption (Figure S12). After optimizing the capture and detection conditions (Figure S13), all other detection conditions followed commercially available antibody detection kit so that NApt ELISA, NAb ELISA, and CNApt ELISA could be scientifically and systematically compared under the same conditions (Figure 4a). For example, the incubation time of NP and horseradish peroxidase (HRP)-labeled reporter (HRP-SA or HRP-labeled secondary antibody) were the same. The capture probes in aptamer-based ELISA and antibody-based ELISA were all precoated in the plates. The total time cost is about 2.5 h starting from the incubation of NP.

We next challenged the detection of NP in 10% fetal bovine serum (FBS) with CNApt ELISA by using NAb ELISA and NApt ELISA as control. LOD was determined as three standard deviations above the background.^43^ As shown in Figure 4b, LOD of NApt ELISA (724 pg/mL) was about 15-fold higher than that of NAb ELISA (44.1 pg/mL) in ordinary PBS buffer (PBS, 0.1% Tween-20, pH 7.4). This result could be explained by the following two fact situations. First, antibodies mAb/rAb presented higher binding affinity to NP than aptamers B15/B61 (Figure 3a, Figure S7). Second, the bivalent, stable, and rigid structure made antibodies more tolerant to environmental changes, as well as more resistant to washing procedure, than conformation-labile aptamers (Figure 3b,c).^44,45^ However, the LOD of CNApt ELISA (40.1 pg/mL) was 18 times as sensitive as NApt ELISA and compared to that of NAb ELISA because CNApts immensely enhanced the binding affinity of aptamers and the ability of aptamer–protein complexes to resist washing.

To further highlight the advantages of covalent aptamer, a harsh washing buffer (2× saline sodium citrate buffer, 10% formamide, 1% tween-20, pH 7.4), which was usually used in FISH (fluorescence in situ hybridization) assay to exclude background noise,^46^ was adopted in this study. After harsh washing, the LODs of NAb ELISA and NApt ELISA were increased to 70.0 and 2015 pg/mL, respectively (Figure 4c). In comparison, the LOD of CNApt ELISA was decreased to 8.70 pg/mL, which was a ∼4.6-fold improvement compared to that of CNApt ELISA under ordinary washing condition (Figure 4c). These changes were probably because covalent aptamer-based ELISA had a better ability to resist the harsh washing, which not only removes nonspecific binding but also partially impairs the specific interaction between antibody/aptamer and target protein. For example, after harsh washing, the saturated signals of CNApt ELISA, NApt ELISA, and NAb ELISA were decreased from ∼3.0 to ∼2.5, ∼3.0 to ∼2.0, and ∼2.9 to ∼0.8, respectively (Figure 4b,c). Therefore, after multiple harsh washing, CNApt ELISA performed 8 times and 232 times as sensitive as NAb ELISA and NApt ELISA, respectively. In addition, the linear dynamic range of NApt ELISA shifted up 3 times (from 729 to 19683 pg/mL to 2187–59049 pg/mL) because the harsh washing impaired the interaction between aptamer and target protein (Figure 4d,e). However, the linear dynamic range of CNApt ELISA expanded 9 times (from 81 to 2187 pg/mL to 81–19683 pg/mL), which got broader than NAb ELISA after harsh washing (Figure 4d,e). Therefore, the comparison of CNApt ELISA and classic NAb ELISA showed our covalent aptamer strategy facilitated more sensitive LOD, broader detection range and less cost (Table S3).

Given the compatibility of aptamer and various nucleic acid-based signal amplification strategies, RCA (rolling cycle amplification) was adopted to further expand the robustness, reliability, and applicability of covalent aptamer-based ELISA. As shown in Figure S14a, the detection aptamer NHS-61 was lengthened with a 20-nt DNA sequence, which could hybridize with a circular DNA template (Figure S14b) and then execute the RCA reaction. The RCA reaction was monitored by agarose gel electrophoresis and a highly efficient, micron-sized RCA product was obtained (Figure S14c,d). After the polymerization times were optimized (Figure S14e), we tested the ELISA detection performance in 10% FBS by harsh washing. The introduction of RCA made the LOD of CNApt ELISA and NApt ELISA decrease to ∼102 times and ∼85 times, respectively (Figure S15a). As shown in Figure S15b, both of their linear ranges shifted down 81 times after integrating RCA method and the linear range of CNApt-RCA ELISA (0.111–27 pg/mL) kept 9 times as broad as NApt-RCA ELISA (27–729 pg/mL). These results attested the robustness of CRApt-RCA ELISA under harsh washing. Of note, the introduction of RCA only cost another 1 h, about 3.5 h.

The covalent aptamer-based ELISA displayed much lower LOD (8.70 pg/mL) than aptamer-based proximity-dependent qPCR amplification assay with a LOD of 37.5 pg/mL or commercial lateral flow immunoassay with a LOD of 0.65 ng/mL.^47,48^ To further demonstrate its specificity, NP, along with spike protein (SP) of SARS-CoV-2, lysozyme and HSA was measured by CNApt ELISA and NApt ELISA in above-mentioned two washing ways. Under ordinary washing, CNApt ELISA demonstrated 12.3–40.0 times selectivity on the detection of NP than these nontargeted proteins; however, NApt ELISA only showed 4.7–8.4 times selectivity on the detection of NP than these nontargeted proteins (Figure 4f). Under harsh washing, the selectivity of CNApt ELISA between NP and nontargeted proteins increased to 61.9–138.4 times, but the selectivity of NApt ELISA between NP and nontargeted proteins decreased to 2.1–2.6 times. All these results clearly reveal that the sensitivity and the specificity enable the covalent aptamer-based ELISA hold a promising prospect in detection application.

### Binding Capacities of Covalent RBD Aptamer

**Figure 5:**
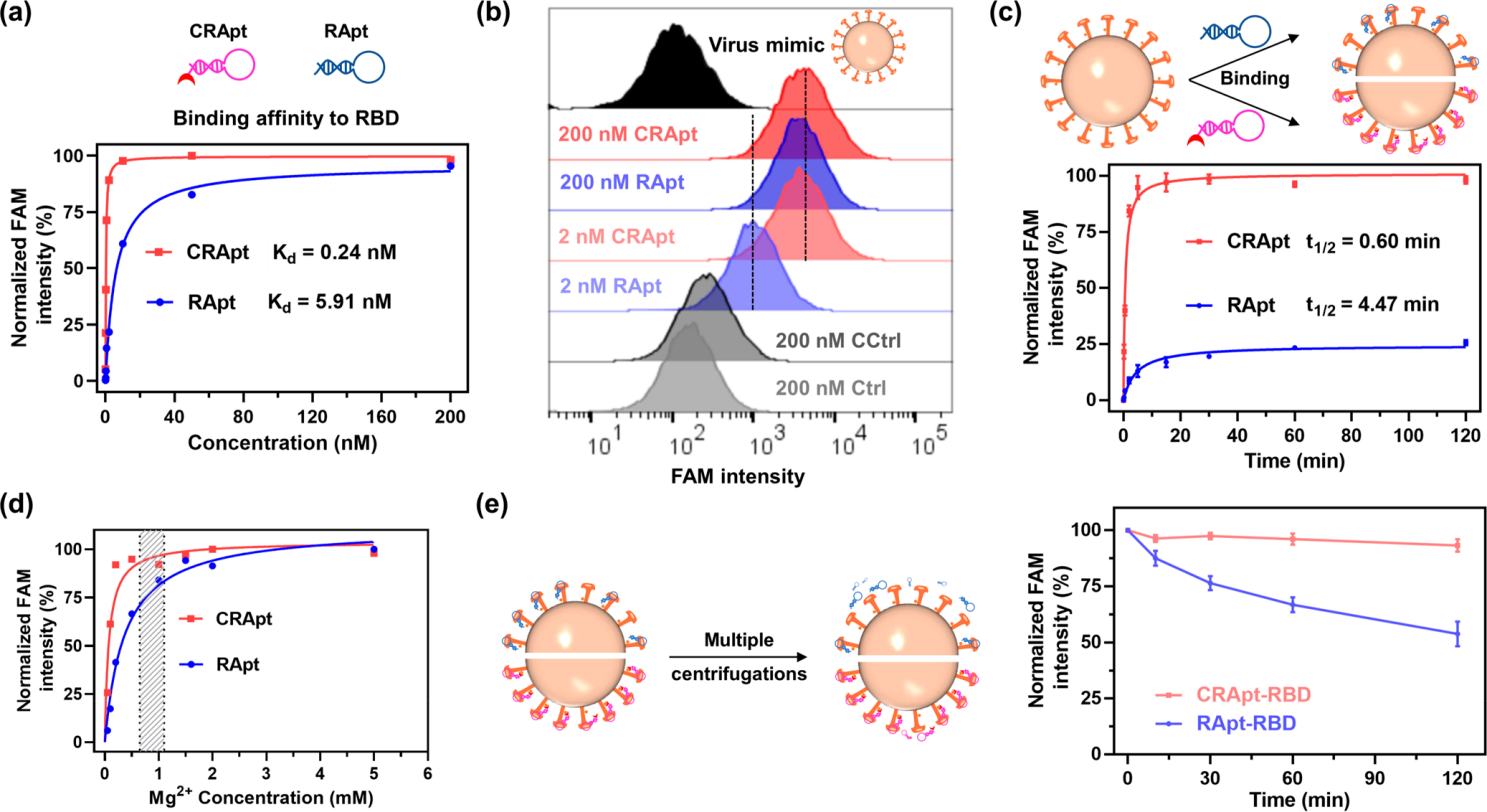
Covalent cross-link enabled RApt to improve various binding properties and decrease less detachment. (a) Binding affinity analysis of CRApt and RApt against RBD protein at room temperature (RT). (b) Representative binding events of RBD protein in flow cytometry assay. (c) Binding kinetics analysis of 2 nM CRApt and RApt. All data are mean ± s.d., *n* = 2. (d) Binding ability analysis of 200 nM CRApt and RApt under different Mg^2+^ concentrations. (e) Detachment situations of 2 nM CRApt and RApt from protein bound complexes after incubation in 10% FBS at 37 °C and multiple centrifugations. All data are mean ± s.d., *n* = 2.

As demonstrated in our study, we selected NHS-labeled neutralizing RApt (CRApt) to enhance antagonistic function of RBD protein by using noncovalent neutralizing RApt and neutralizing antibody (RAb) as controls. Before the neutralization analysis, the binding performance of NHS-RApt, RApt and RAb on RBD protein was analyzed by flow cytometry assay with using RBD protein-coated nickel microbead as a virus mimic. The binding affinity of CRApt (0.24 nM) showed 25 times as strong as RApt (5.91 nM) and was similar strength as RAb (0.33 nM), resulting in that 2 nM CRApt performed similar binding ability as 200 nM RApt (Figure 5a,b, Figure S16). It is worth noting that the binding half-time of CRApt, RApt, and RAb on virus mimics was 0.60, 4.47, and 0.80 min, respectively (Figure 5c, Figure S17). Besides, 200 nM covalent control DNA (CCtrl) had weak binding on virus mimic than noncovalent Ctrl (Figure 5b), and 200 nM CRApt also displayed weak binding on NP-coated nickel microbead (Figure S18). And the analysis of the effect of Mg^2+^ concentration on the binding of NHS-RApt to RBD revealed that CRApt, rather than RApt, presented saturated binding strength even under physiologic Mg^2+^ concentration range (0.65–1.10 mM)^42^ (Figure 5d). These results demonstrated that NHS-RApt could strongly, specifically, and rapidly bind to RBD even at physiological environment with low divalent metal ions.

The stability of the probe–RBD complexes would affect the neutralization efficacy of RBD-targeted probes; therefore, we analyzed the dissociation of aptamer–RBD complexes and antibody–RBD complexes by measuring the fluorescence signal of virus mimics with flow cytometry after the complexes were incubated in 10% FBS at 37 °C for different time and then centrifuged twice. After four tests, CRApt detached ∼7% from RBD-bound complexes while RAb and RApt detached ∼14% and ∼46% from probe–protein complexes in the time point of 120 min (Figure 5e, Figure S18). The antinuclease degradation analysis of the aptamer–protein complexes in 10% FBS at 37 °C was also executed. We found CRApt–RBD complexes and RApt–RBD complexes could be stable for 8 h (Figure S19). The long stability suggested the detachment test was not disturbed by serum-mediated degradation and protein-bound aptamers could improve their biostability. Then we studied the detailed cross-link sites by LC-MS/MS experiment and molecular docking simulation. LC-MS/MS analysis showed only K-417 could not been recognized and cleaved by trypsin because it had been modified by CRApt. Molecular docking simulation further provided the structural information for the cross-link that the distance between the nitrogen atom in primary amino group of lysine residue (K-417) in RBD and the carbon atom in the carbonyl group next to NHS group was 2.9 Å (Figure S20). K417 is just a linkage amino acid bridging the interaction RBD protein and ACE2 receptor.^49^ So the docking result revealed CRApt could seriously inhibit the interaction of RBD.

### Covalent Aptamer-Mediated Inhibition of Interaction between RBD and ACE2 under Different Environments

**Figure 6:**
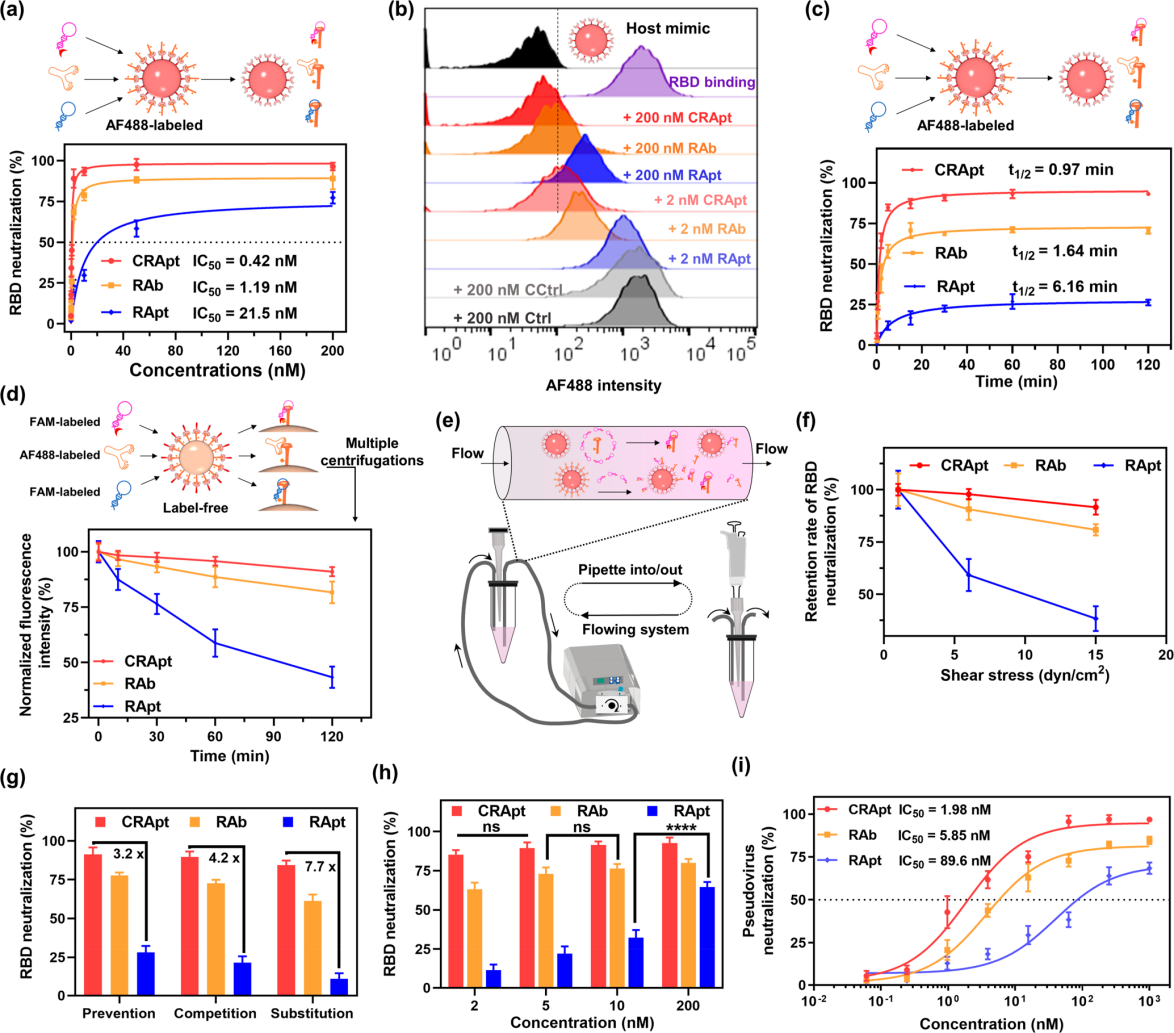
Covalent cross-link strategy improved RApt-mediated functional blocking ability under different tough environments. (a) RBD neutralization analysis of different concentrations of RBD probes in 10% FBS at 37 °C. (b) Representative flow cytometry analysis of RBD neutralization after different RBD probes blocked RBD-preincubated host mimic. RBD protein was labeled by AF488 and host mimic was ACE2-coated nickel microbead. (c) Neutralization kinetics analysis of 2 nM RBD probes in 10% FBS at 37 °C. (d) Detachment situations of 2 nM RBD probes blocking the ACE2-virus mimic interaction. The detachment assay was tested after incubation in 10% FBS at 37 °C and multiple centrifugations. (e) Schematic illustration of RBD neutralization assay in blood vessel-mimic flowing circulation system with 10% FBS. (f) Retention rate analysis of neutralization of 2 nM RBD probes under different flowing speed-mediated shear stresses. (g) Neutralization analysis of 2 nM RBD probes in different blocking ways under the circulation system. (h) Substitution-typed RBD neutralization analysis of different concentrations of RBD probes under the circulation system. (i) Pseudovirus neutralization analysis of different RBD probes against ACE2-expressed 293T host cells from infecting luciferase reporter-carried pesudovirus. All data are mean ± s.d., *n* = 2 in all statistical analysis. Statistical significance: ****P < 0.0001, ns means no significant difference.

On the basis of the improvement of binding performance of CRApt, we then evaluated the neutralization ability of CRApt under different conditions. The neutralization ability of CRApt, RAb, and RApt was first analyzed by measuring their ability to neutralize the interaction between AF488-labeled (Alexa Fluor-488) RBD and host mimic, ACE2-coated nickel microbead, in 10% FBS at 37 °C. The IC_50_ value of CRApt neutralization ability was 0.42 nM, 1.8 times and 50.2 times lower than RAb and RApt, respectively (Figure 6a). The neutralization ability of 2 nM CRApt could achieve 90% of saturated inhibition of interaction between RBD and host mimic, comparable to that of 200 nM RAb (Figure 6a,b). 200 nM Ctrl and CCtrl DNA showed no apparent neutralization ability, which indicated again NHS-conjugated aptamer showed favorable specificity (Figure 6b). Furthermore, the half saturated inhibitory time (*t*_1/2_) of CRApt, RAb, and RApt was 0.97, 1.64, and 6.16 min at the concentrations of 2 nM respectively (Figure 6c). Their saturated inhibition rates of interaction between RBD and host mimic were 93%, 73% and 28% respectively (Figure 6c, Figure S21). The half full inhibitory time (IT_50_) of 200 nM CRApt, RAb, and RApt was 0.87, 1.37, and 3.97 min (Figure S22). These results indicated that CRApt could rapidly and potently neutralize RBD. Besides, we also tested the binding ability and the neutralization efficiency of CRApt against Omicron variant RBD protein of SARS-CoV-2. Unfortunately, there is no covalent cross-linking and enhanced neutralization ability of CRApt against Omicron variant RBD protein, although CRApt could bind to it at the binding affinity of 14.7 nM (Figure S23). The reason may be that there is no reactive lysine residue of Omicron variant RBD protein near the binding pocket because the Omicron variant has over 30 mutations including K417N.

Next, we tested the neutralization ability of CRApt to block the RBD-ACE2 interaction under different tough environments. The more stable the ligand–virus mimic complexes, the more potent the neutralization ability of the ligands. After incubation in 10% FBS for 120 min followed by multiple centrifugations, only 8% of CRApt–virus mimic complexes was disassociated; however, the disassociation rates were 18% and 57% for RAb–virus mimic complexes and RApt–virus mimic complexes, respectively (Figure 6d). RBD-neutralizing ability of CRApt, RAb and RApt was also tested in a blood vessel-mimicking flowing circulation system, which simulates the fluid shear stress in vivo (Figure 6e, Figure S24). Normal blood shear stress of human body is 1–6 dyn/cm^2^ and some arterial blood vessels can cause shear stress of 15 dyn/cm^2^.^50^ When the fluid shear stress was increased from 1 dyn/cm^2^ to 15 dyn/cm^2^, the RBD-neutralizing ability of CRApt only decreased 8%; however, the RBD-neutralizing ability of RAb and RApt decreased 19% and 62%, respectively (Figure 6f, Figure S25a). Above all, the results revealed that the covalent aptamers hold potential to block the interaction between RBD-ACE2 in physiological environment.

Then the neutralization efficiency of NHS-RApt, RAb and RApt in prevention model, competition model and substitution model were tested under shear stress of 15 dyn/cm^2^, respectively. In the prevention model, the neutralizers were first incubated with RBD, and then host mimics were added. In the competition model, the neutralizers, RBD, and the host mimics were incubated simultaneously. In the substitution model, RBD was first incubated with host mimics, and then the neutralizers were added. In all three models, the covalent aptamer demonstrated the best neutralizing efficacy than commercial antibody and noncovalent aptamer. For example, in the harshest environment (substitution module under shear stress of 15 dyn/cm^2^), the neutralizing efficacy of NHS-RApt was up to ∼85%; however, the neutralizing efficacy of RAb and RApt was ∼60% and ∼10%, respectively (Figure 6g, Figure S25 a,b). Our results further demonstrated that the lowest concentration to achieve the saturated neutralization was 2, 5, and 200 nM for NHS-RApt, RAb, and RApt, respectively (Figure 6h, Figure S25c). Neutralization kinetics analysis revealed that the covalent aptamer could rapidly achieve the saturated neutralization in 5 min, while RAb and RApt needed 15 min and at least 30 min to achieve their saturated neutralization, respectively (Figure S25d). To further verify the neutralization efficiency in more realistic environment, we tested the neutralization ability of CRApt, RAb and RApt to block the infection of luciferase reporter-carried pseudoviruses on ACE2-expressed 293T host cells. The IC_50_ value of the pseudovirus neutralization ability of the covalent aptamer was 1.98 nM, 2.0 times and 44.2 times lower than those of RAb and the noncovalent aptamer, respectively. Their saturated inhibition rates of interaction between pseudoviruses and host cells were 95%, 81%, and 70%, respectively (Figure 6i). These data demonstrate that our covalent aptamer strategy holds a promising prospect in inhibiting the infection of SARS-CoV-2 in realistic environment.

## Conclusion

In summary, by discussing and selecting suitable covalent tags, we reported covalent aptamers that are able to efficiently, specifically, and covalently cross-link with target protein by aptamer-guided proximity-driven “click” bioconjugation, coupled with conversion of noncovalent aptamer–protein complexes into covalent complexes. The resulting aptamer–protein covalent complexes can resist various environmental interferences, especially multiple harsh washings and shear stress.

As a practical proof-of-concept, first we realized the ultrasensitive detection of NP of SARS-CoV-2 by designing a covalent aptamers-based sandwich ELISA under harsh washing, about 8 times as sensitive as the commercially available antibody-based sandwich ELISA. Benefitting from the compatibility with nucleic acid-based amplification, the detection sensitivity could be further improved by 2 orders of magnitude (from 8.70 pg/mL to 0.0859 pg/mL). Besides, we also verified the robustness and reliability of the covalent aptamer strategy in various neutralization measurements of RBD protein of SARS-CoV-2. Two nM covalent aptamer could obtain stronger blocking efficiency than 200 nM traditional aptamer and antibody under tough physiological environment with shear stress. It is worth noting that our covalent aptamers can only detect or neutralize present NP and RBD protein of wild-type SARS-CoV-2, respectively. However, it can be anticipated that covalent aptamers against SARS-CoV-2 variants can be designed if more structural information could be obtained from aptamer and Omicron variant RBD protein. In a word, based on proximity-driven covalent conjugation strategy, our reasonably designed covalent aptamer strategy can inspire further development and even industrial applications of aptamers in various fields of life sciences.

## Supporting Information Available

Reagents; detailed experimental procedures; ^1^H NMR spectrum; ^13^C NMR spectrum; DNA sequences for this study; MS analysis of NHS-aptamer and additional characterization of covalent aptamers (PDF)
