# Supplementary material for: Robust Covalent Aptamer Strategy Enables Sensitive Detection and Enhanced Inhibition of SARS-CoV-2 Proteins
Source: ACS Cent Sci. 2023 Jan 2;9(1):72–83. doi: 10.1021/acscentsci.2c01263 (PMC9881204; doi:10.1021/acscentsci.2c01263)
Supplement: Supplementary file 1 — oc2c01263_si_001.pdf [file oc2c01263_si_001.pdf]

Supporting Information for

Robust Covalent Aptamer Strategy Enables Sensitive Detection and Enhanced Inhibition of SARS-CoV-2 Proteins

Dan Wang,[1,2,4‡] Jing Zhang,[1,‡] Zhiyong Huang,[1,‡] Yuhang Yang,[1] Ting Fu,[2] Yu Yang,[3] Yifan Lyu,[1] Jianhui Jiang,[1] Liping Qiu,[1] Zehui Cao,[3] Xiaobing Zhang,[1] Qimin You,[2,5] Yuankui Lin,[2,5] Zilong Zhao[1,*] and Weihong Tan[1,2,3,*]

[1]Molecular Science and Biomedicine Laboratory (MBL), State Key Laboratory of Chemo/Biosensing and Chemometrics, College of Chemistry and Chemical Engineering, Aptamer Engineering Center of Hunan Province, Hunan University, Changsha, Hunan 410082, China

[2]Zhejiang Cancer Hospital, Hangzhou Institute of Medicine (HIM), Chinese Academy of Sciences, Hangzhou, Zhejiang 310022, China

[3]Institute of Molecular Medicine (IMM), Renji Hospital, Shanghai Jiao Tong University School of Medicine and College of Chemistry and Chemical Engineering, Shanghai Jiao Tong University, Shanghai 200240, China

[4]LIMES Chemical Biology Unit, Universität Bonn, 53121 Bonn, Germany

[5]Ustar Biotechnologies (Hangzhou) Ltd., Hangzhou, Zhejiang 310053, China

*To whom correspondence should be addressed. Email: zlzhao@hnu.edu.cn, tan@hnu.edu.cn

Outline

1. Materials and Methods 3
2. Supporting Figures 7
3. Supporting Tables 32
4. References 36

1. Materials and Methods

1.1 General materials.

All chemicals for organic synthesis and purification were purchased from Energy Chemical. Streptavidin-coated magnetic beads for biotin-labeled conjugation and Ni beads for His-tagged conjugation were from GE Healthcare (USA). ELISA coating buffer, crystal violet and AF488–conjugated Goat Anti-Mouse IgG (H+L) were obtained from Solarbio (Beijing). Phi29 DNA polymerase was purchased from Sangon Biotech. SARS-CoV-2 nucleocapsid protein (His tag), His tag-labeled RBD and mFc-labeled wild-typed RBD and RBD Omicron variant protein, SARS-CoV-2 Nucleocapsid Detection ELISA Kit (KIT40588), mouse antibody MAb (label free and HRP-labeled) and rabbit antibody RAb (label free and HRP-labeled) were obtained from Sino Biological Inc. Recombinant IL-2 protein was purchased from Beijing T&L Biological Technology Co.,Ltd. His tag and biotin-labeled ACE2 protein was purchased from AtaGenix Laboratories Co., Ltd (Wuhan). ApexHF HS DNA Polymerase FS was purchased from Accurate Biotechnology (Hunan) Co.,Ltd. Super GelRed and agarose were obtained from US Everbright® Inc. HRP-labeled SA and TMB were obtained from Beyotime Biotech. ELISA plates were purchased from NEST Biotechnology. Binding buffer (BB) was ordinary PBS supplemented with 5 mM $MgCl_2$, 300 mM NaCl, 400 nM BLib and 2 μg/mL salmon sperm DNA. Washing buffer (WB) was PBS supplemented with 5 mM $MgCl_2$ and 0.1% Tween-20. All oligonucleotides in Table S1 were purchased from Sangon Biotech and then dissolved in Milli-Q ddH_2O for synthesis or other experiments. Other reagents and materials were obtained from Sigma unless otherwise specified.

1.2 Synthesis of azide-labeled sulfuryl fluoride (SF) warhead.

4-(Bromomethyl)benzenesulfonyl fluoride (40 μmol) and sodium azide (38 μmol) were mixed in 2 mL DMSO. The reaction mixture was vortexed for 30 minutes at room temperature, then mixed with cold water (0.5 mL) and extracted with ethyl acetate (1 mL). The collected organic phase was washed with saturated $NaHCO_3$ (0.5 mL × 2), dried over Na_2SO_4, and evaporated to yield a yellow solid pure product (yield 56%), i.e. SF warhead.

1.3 Synthesis of covalent tags-conjugated DNAs.

To obtain SF-conjugated aptamers, DBCO-labeled DNAs (10 nmol, soluble in 100 μL water) and SF warhead (1 μmol, soluble in 100 μL DMSO, 100 eq.) were mixed and then shaken at room temperature for 30 min. To obtain NHS-conjugated aptamers, amino-labeled DNAs (10 nmol, soluble in 100 μL water) and disuccinimidyl suberate (DSS, 1.4 mg, soluble in 100 μL DMSO, 400 eq.) were mixed with 1 μL triethylamine and then shaken at room temperature for 30 min. To obtain acrylamide-conjugated aptamers, amino-labeled DNAs (10 nmol, soluble in 100 μL water) and 2,5-dioxopyrrolidin-1-yl acrylate (4 μmol, soluble in 100 μL DMSO, 400 eq.) were mixed 1 μL triethylamine and then shaken at room temperature for 30 min. All the crude products of covalent tags-conjugated DNA were synthesized in this way and purified as follows.

3 M NaCl buffer (20 μL) and cold ethanol (500 μL) were added in the crude products and then frozen at -20 °C for 30 min. Then the DNA precipitates could be collected by centrifugation for 30 minutes (4 °C, 14000 g). The precipitates were then dissolved in 200 μL 0.1 M TEAA and purified by reversed-phase HPLC (0-30 % acetonitrile in 0.1 M TEAA over 40 minutes, flow rate= 1 mL/min) on SPD-M20A (SHIMADZU, Japan). Specially, to stabilize the separated NHS ester-modified DNA, an equivalent amount of a 2% (v/v) trifluoroacetic acid (TFA) solution was added to reduce the hydrolysis rate of NHS ester. Finally, the product was vacuum-dried and verified by mass spectra (Table S2).

1.4 Molecular simulation of protein docking.

The molecular simulation of NHS-labeled aptamer (see figure below, including NHS-Apt15, NHS-Apt61 and NHS-RApt) docking with target protein (PDB of NP: 6WZO; PDB of RBD protein: 6VSB) was executed with Gromacs software (Yiran Biotech, Baoding, China).

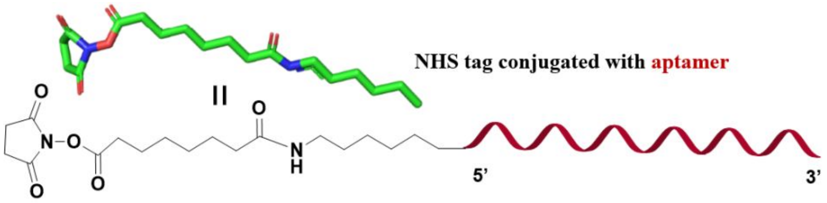

1.5 LC-MS/MS analysis

After collecting the crosslinking bands of CApts and POIs by reducing SDS-PAGE, the bands were dissolved and extracted for trypsin-mediated digesting to abundant peptides which underwent LC-MS/MS assay (Sangon Biotech). Sequence information from MS/MS data was processed by converting the .raw files into a merged file (.mgf) using PEAKS Studio software. One missed cleavage (N-terminal of arginine or lysine) for the trypsin was permitted. If peptides contained a lysine which could not been digested without missed, they will be modified by covalent aptamers.

1.6 Surface plasmon resonance (SPR) assays.

First, 2 μg His-tagged NP was immobilized on the carboxymethylated sensor chip (CM5 chip) with pH 5.5 buffer. Then ethanolamine-HCl was used to deactivate unreacted carboxyl groups. Binding buffer served as running buffer, 1 M NaCl served as regeneration buffer of aptamer-based SPR assays and Glycine-HCl (pH 2.0) served as regeneration buffer of antibody-based SPR assays. The running condition was set at a 10 μL/min flow rate, 25 °C, 3 min association time, and 3 min dissociation time. His tag signal was used as background subtraction. A series of sensorgrams of designed concentrations of aptamers and antibodies was tested and then analyzed with GraphPad Prism 8 software to obtain association rate constant K_{on}, dissociation rate constant K_{off} and equilibrium dissociation constant K_d.

1.7 Binding specificity investigation by fluorescence-based direct ELISA assay.

First, 10 μg/mL NP, lysozyme, SA and 1 mg/mL HSA were precoated in ELISA wells overnight, respectively. Then 20 nM FAM-labeled DNAs (Apt15, Apt61, NHS-Apt15, NHS-Apt61, Ctrl and NHS-Ctrl) were incubated correspondingly for 1 h at room temperature after blocking (salmon sperm DNA, NaCl) and washing (WB) the ELISA plates. After washing 3 x, these wells were added to 100 μL PBS to test the fluorescence value using a Synergy 2 Multi-Mode Microplate Reader (Bio-Tek). Meanwhile, the fluorescence value of FAM-labeled Apt15 of different concentrations soluble in ELISA wells was tested in order to construct an external standard quantitative curve (linear range, 0.0625 nM to 32 nM). Based on the quantitative equation, real binding quantity of FAM-DNA could be calculated.

1.8 Signal-background-ratio (SBR) measurements to investigate optimal or superior group.

The optimization of binding buffer was based on PBS buffer reported in a previous work.[1] Aptamer pairs, the concentrations of salmon sperm DNA, biotin-labeled short DNA (BLib) and NaCl and the incubation time of HRP-SA were all tested under corresponding concentrations of NP-based sandwich ELISA assay. The incubation methods of capture aptamer (FAM-labeled B15) and the SBR measurements of washing times (three minutes every time) were tested by NP-based direct ELISA assay. The optimization of the amplification condition (polymerization time of phi29 DNA polymerase) was tested by 1 pg/mL NP-based sandwich ELISA assay. The detachment analysis of NP probes was conducted under one to eight washing (3 min every time) after HSA-labeled SA or HSA-labeled secondary antibody was added to bind with NP probes. All SBRs were calculated as OD_{450} (sample concentration)/ OD_{450} (zero concentration), where OD_{450} was subtracted from the TMB background value (0.0565).

1.9 Construct of RCA reaction.

First, to prepare a circular template, 8 μL 100 μM padlock probe, 8 μL 100 μM ligation probe, 2 μL 350 U/μL T4 DNA ligase, and 2 μL 10x T4 DNA ligase buffer were mixed at 16 °C for 30 min. Then 5 U Exonuclease I and 10 U Exonuclease III were added and incubated at 37 °C for 1 h before deactivating at 70 °C for 10 min, resulting in a purified circular template. Then, 10 nM NHS-61,10 nM DNA circular template, 1 U phi29 DNA polymerase and 1 μM dNTPs were mixed in phi29 buffer for 30 min. Finally, 100 nM cDNA was treated for 15 min to obtain the final RCA product. All above

procedures and products were monitored by agarose gel electrophoresis and AFM imaging.

1.10 Gel electrophoresis.

To monitor the RCA procedure described previously, 3% agarose gel electrophoresis (AGE) was carried out in 1× TBE/Mg (90 mM Tris, 9 mM boric acid, 1 mM EDTA, 5 mM Mg^{2+}, pH 8.0) at 110 V for 30-60 min. To investigate proximity-driven covalent aptamer-based bioconjugation, NP samples were treated with 0.05% NP-40, 1% SDS and 400 mM NaCl to remove the positive charge and possible nonspecific electrostatic adsorption.[2] The corresponding treated samples were run with 10% native polyacrylamide gel electrophoresis (PAGE, Lianshuo Biotech) or urea-based denatured PAGE in 1× TBE/Mg or 1x TBE at 110 V for 30-75 min. To investigate the covalent crosslink of three covalent aptamers by reducing SDS-PAGE, firstly POI was mixed with corresponding covalent aptamers in HEPES buffer at room temperature for designated time. For the kinetics measurements, the reaction need be quenched by 50 mM glycine. Then 10% SDS-PAGE was carried out in MOPS SDS running buffer under NuPAGE Bis-Tris system. All gels were stained with Super GelRed or Coomassie Blue and imaged using a Bio-Rad ChemiDoc XRS System.

1.11 AFM imaging

Initially, 20 μL 1x TBE/Mg containing 30 mM Ni^{2+} were added on the surface of freshly cleaved mica to enhance the adhesion of DNA samples. Afterwards, 10 μL 10 nM purified RCA product was deposited for 5 min to allow for adsorption. Finally, the mica was rinsed with ultrapure water three times and dried in the nitrogen atmosphere. Atomic force microscopy of samples was observed on a Multimode 8 (Bioscope system, Bruker, USA) using Scan Assist mode in atmosphere.

1.12 Sensitivity and specificity tests of antibody-based and aptamer-based ELISA.

To compare CNApt ELISA, NApt ELISA, NAb ELISA at the same level, most operation procedures were kept in the same or similar way. To be specific, for commercial antibody sandwich ELISA, the ELISA assay followed the manufacturer's protocol with modifications. 100 μL NP of different concentration gradients dissolved in BB or supplemented with 10% FBS (including blocking reagents), were added in the ELISA wells precoated with MAb. After incubation for 1 h, the wells were washed three times and then Rabbit antibody (RAb) treated for 0.5 h. Then HRP-labeled secondary antibody was incubated for 15 min after washing the unbinding RAb. Afterwards, 100 μL TMB were added and incubated for 15 min after washing 3 x. Finally, 50 μL H_2SO_4 at the concentration of 2 M were added to stop the enzyme-catalyzed reaction, followed by collecting the absorbance at 450 nm using a Synergy 2 Multi-Mode Microplate Reader (Bio-Tek).

For noncovalent or covalent aptamer-based sandwich ELISA, 100 μL NP of different concentration gradients dissolved in BB or supplemented with 10% FBS (including blocking reagents), were added in the precoated ELISA wells where SA-coated ELISA plate was preincubated with 100 μL B15 or NHS-B15 at the concentration of 20 nM at 4 °C overnight. After incubation for 1 h, the wells were washed three times and then 10 nM B61 or NHS-B61 treated for 0.5 h. Then HRP-SA was incubated for 15 min after washing the unbinding B61 or NHS-B61. The latter procedures were the same as those previously indicated in NAb ELISA.

For the noncovalent aptamer or covalent aptamer-based RCA ELISA, the incubation methods of B15 (or NHS-B15), NP and Apt61 (or NHS-61) were the same as those previously described. After washing, 10 nM DNA circular template, 10 U phi29 DNA polymerase and 10 μM dNTPs were incubated for 30 min. Then 100 nM HRP-cDNA were treated for 15 min after washing 3 x. The latter procedures were the same as those previously described in NAb ELISA.

For NP specificity test of CNApt under ordinary washing and harsh washing, 1 ng/mL NP, SP and lysozyme, 100 ng/mL HSA were used to test the SBR value in the NApt or CNApt sandwich ELISA. The corresponding procedures were the same as those previously described.

1.13 Flow cytometry assays.

All flow cytometry assays were finished in DxP Athena (US Cytek Biosciences, Inc.). To test the binding affinities of three RBD neutralizers (CRApt, RApt, RAb) and control DNAs (Ctrl, CCtrl) against RBD protein or Omicron variant RBD

protein under binding buffer or under different concentrations of Mg^{2+} buffer, FAM-labeled DNAs of different concentrations were incubated with His tagged-RBD-precoated Ni beads (virus mimic) for 1 h at RT. Then all samples were centrifuged to wash 3 x, 2 min every time. Specially, AF488-conjugated secondary antibody was used to label RAb group for 0.5 h and then washing 3 x. Finally, all samples from above groups were tested in the flow cytometer.

To test binding kinetics of RBD neutralizers, virus mimic was incubated with 2 nM RBD neutralizers for different time periods (10 s~120 min), then all samples were immediately centrifuged to wash 2 x, 2 min every time. Specially, AF488-conjugated secondary antibody was used to label RAb group for 0.5 h and then washing 2 x. Finally, all samples from above groups were tested in the flow cytometer.

To test detachment situations of neutralizers from virus mimic, their binding complexes at the timepoint of 120 min were prepared firstly in a previously described way, and then were incubated in 10% FBS at 37 °C for different time periods (10 min~120 min). After treated for different time periods, the binding complexes were centrifuged at 4000 g for twice, 2 min every time. Finally, all samples from above groups were tested in the flow cytometer.

To test various blocking situations of neutralizers between ACE2 protein and RBD, His-tagged ACE2-precoated Ni beads as host mimic was incubated with mFc-tagged RBD protein or Omicron variant RBD protein for 1 h firstly and then AF488-conjugated secondary antibody was used to label the “infected” mFc-tagged RBD protein or Omicron variant RBD protein. As for the neutralization efficiency, neutralization kinetics, detachment situations and other functional blocking characterizations, the corresponding procedures were the same as those previously described in binding measurements. It is worth noting that all neutralization assays were executed in 10% FBS. For the flowing circulation system, a closed-loop circulation was established by connecting a peristaltic pump (INTLLAB Co.) and a 1.5 mL Eppendorf Tube with polyvinyl chloride tubing to mimic the in vivo blood vessel and circulation system.

1.14 Confocal imaging assays.

All confocal imaging assays were finished by using the FV1000-X81 confocal microscope (Olympus, Japan) and all the samples were incubated in 10% FBS. To test blocking efficiency of RBD neutralizers, host mimic was incubated with mFc-tagged RBD protein for 1 h firstly and then AF488-conjugated secondary antibody was used to label the “infected” mFc-tagged RBD protein. Then neutralizers (CRApt, RAb, RApt) at the concentrations of 2 nM were used to substitute the RBD infection in 10% FBS at 37 °C. For another substitution module in the flowing circulation system, different concentrations (2 nM, 5 nM, 10 nM, 200 nM) of neutralizers (CRApt, RAb, RApt) were added in the circulation system to make therapy of the RBD infection in 10% FBS at RT for 30 min under shear stress of 15 dyn/cm^2. Then the Ni beads were collected for flow cytometry assay and confocal imaging assay. The collect procedures were the same as those previously described in binding measurements (centrifuging at 4000 g for twice, 2 min every time).

1.15 Pseudovirus neutralization analysis

To test the pseudovirus neutralization ability of CRApt, RAb and RApt, serial dilutions of these probes in complete DMEM medium were pre-incubated with 5000 TCID50/mL luciferase reporter-carried pseudoviruses per well for 1 h at 37°C, and then the mixtures were added to 104 ACE2-expressed 293T preinoculated cells in 96-well plates. After incubation for 6-hour infection, medium was refreshed and then cells were incubated for another 48 h. Then Bio-Lite Luciferase Assay System (Vazyme Co. Ltd, 100 uL/well) was added. After reaction at room temperature for 5 min, the luciferase activity was measured on a Multimode Microplate Reader (BioTek), and the resulting curves was analyzed by nonlinear regression to calculate the half-maximal inhibitory concentration (IC50) values.

1.16 Statistics.

GraphPad Prism 8 and Origin 2021 were used to show differences between two groups (Student’s *t*-test) or among three or more groups (One-way or Two-way ANOVA with Tukey test). Replicates are three unless otherwise specified. Significant difference is indicated when P value is less than 0.05.

2. Supporting Figures

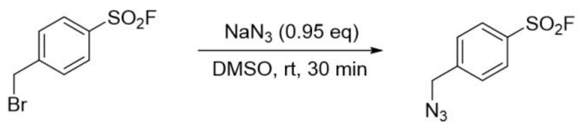

Figure S1. Synthetic scheme of 4-(azidomethyl)benzenesulfonyl fluoride (SF warhead) from 4-(bromomethyl)benzenesulfonyl fluoride.

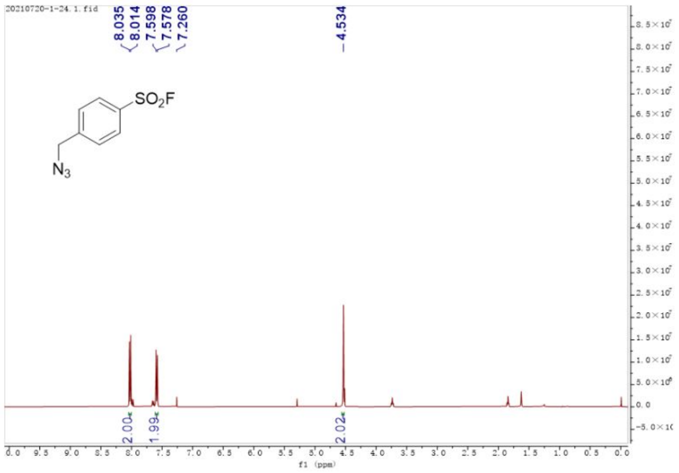

Figure S2. ^{1}H-NMR spectrum of azide-labeled SF warhead.

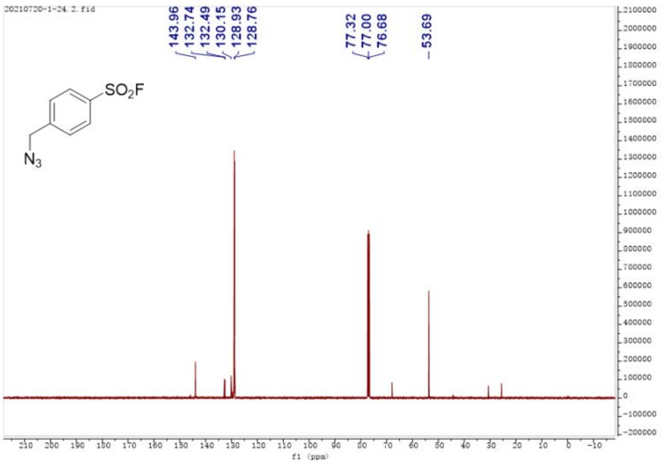

Figure S3. ^{13}C-NMR spectrum of azide-labeled SF warhead.

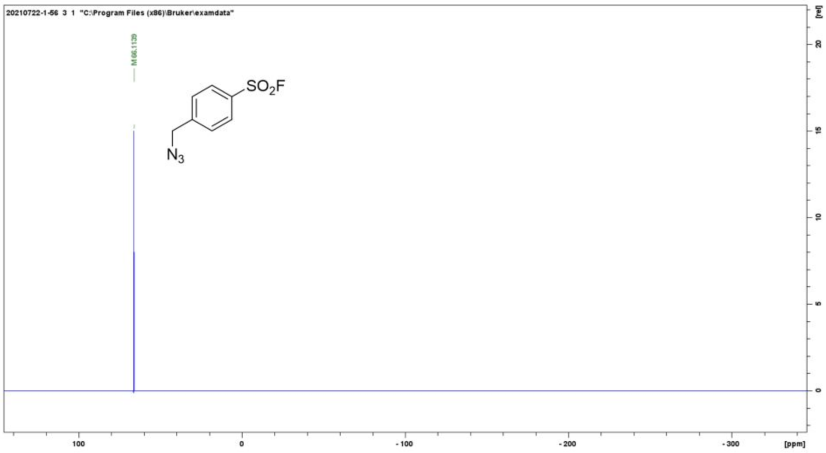

Figure S4. ^{19}F-NMR spectrum of azide-labeled SF warhead.

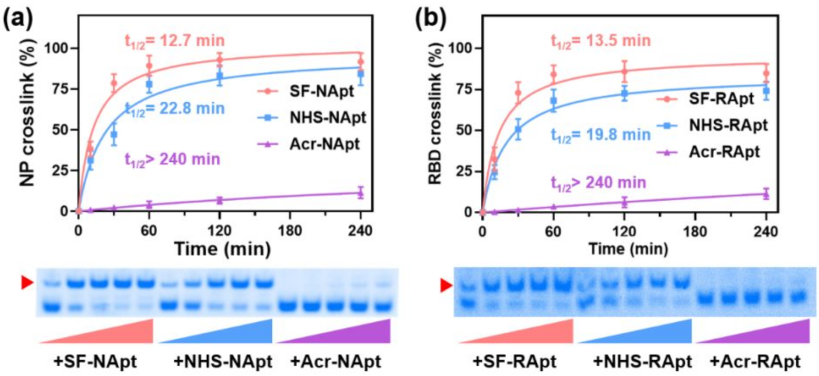

Figure S5. Reducing SDS-PAGE analysis of crosslink kinetics between 400 nM target protein and 2 eq. CApts (protein stained by Coomassie Blue). (a) Reducing SDS-PAGE analysis of crosslink kinetics between 400 nM NP and 2 eq. CNApts. (b) Reducing SDS-PAGE analysis of crosslinking kinetics between 400 nM RBD and 2 eq. CRApts.

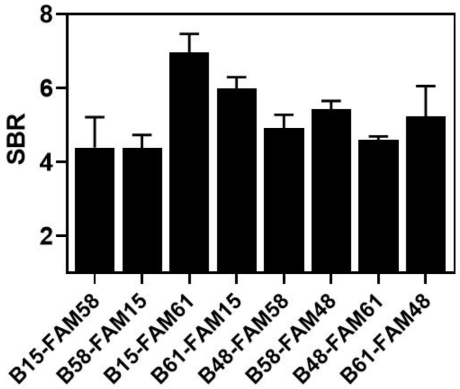

Figure S6. Optimization of aptamer pair (the first one is capture aptamer and the second one is detection aptamer) for sandwich ELISA. Herein SBR was defined as the ratio between sample signal (1 ng/mL NP) and background signal (0 ng/mL NP). Capture aptamers were labeled by biotin to bind to streptavidin-coated plate. Detection aptamers were labeled by FAM to show fluorescence signal.

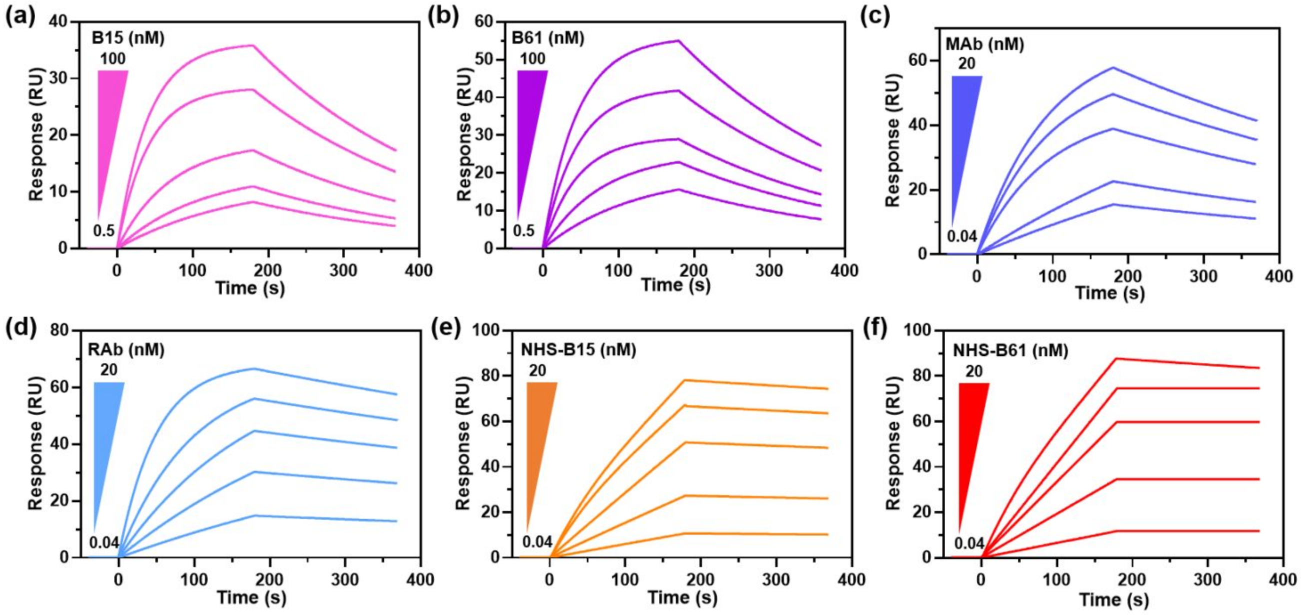

Figure S7. K_d (dissociation equilibrium constant), K_{on} (association rate constant), K_{off} (dissociation rate constant) measurements of NP probes (noncovalent aptamers, antibodies and NHS-conjugated aptamers) by SPR assays. Association and dissociation kinetics of (a) B15, (b) B61, (c) MAb (mouse antibody), (d) RAb (rabbit antibody), (e) NHS-B15 and (f) NHS-B61 by SPR assays. K_d results (Figure 3a) were calculated by analyzing K_{on} and K_{off} from (a-f).

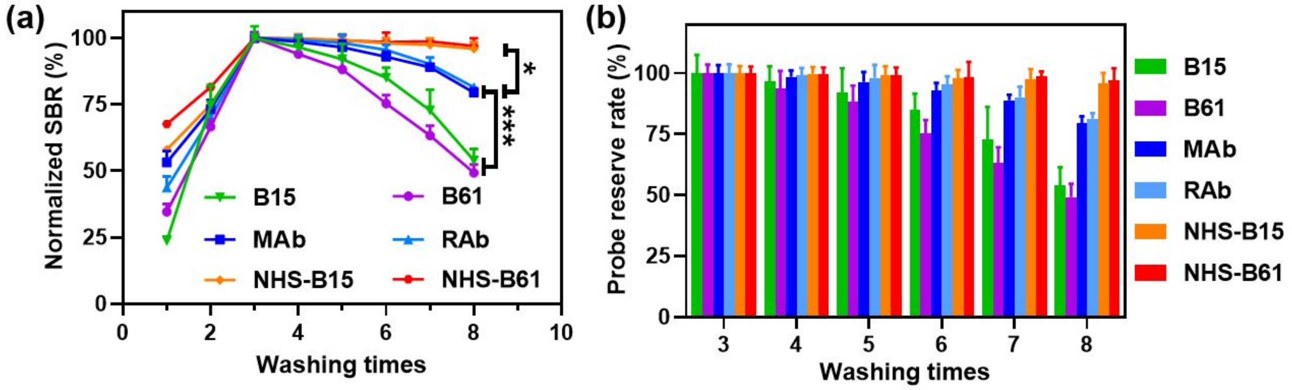

Figure S8. Investigating the reserves of six probes during direct ELISA assays by testing SBR after multiple washings. Every washing cost three minutes. (a) Normalized SBR curve after one to eight washings. (b) Probe reserve rate by considering the SBR value of third washing as 100%. All data are mean ± s.d., n = 2. Statistical significance: ****P* < 0.001, **P* < 0.05.

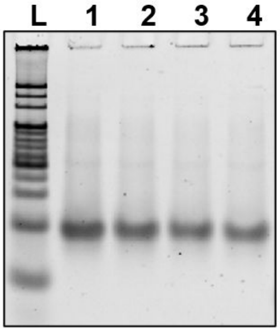

Figure S9. PAGE analysis of control DNA binding with NP. Lane L: 20 bp DNA ladder; lane 1: BCtrl; lane 2: BCtrl+ NP; lane 3: NHS-BCtrl; lane 4: NHS-BCtrl+ NP.

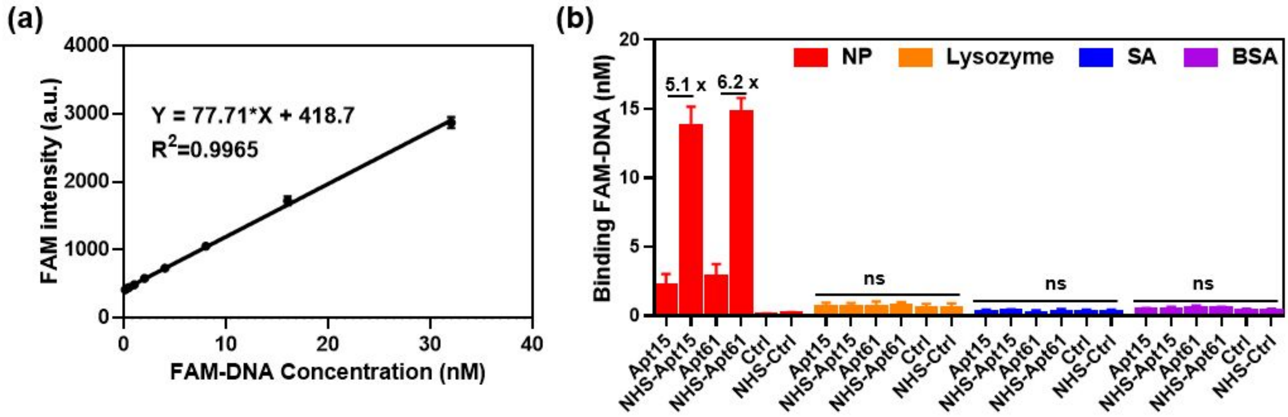

Figure S10. Binding selectivity test of FAM-labeled covalent aptamers by direct ELISA assays. (a) Standard quantitative curve of FAM-labeled DNA (FAM-Apt15). Actual binding concentration of FAM-DNA can be calculated based on this linear equation. (b) Binding quantity of 20 nM FAM-labeled DNA probes in high incubating concentration of 10 μg/mL NP, lysozyme, SA and 1 mg/mL HSA precoated in ELISA wells. All data are mean ± s.d., n = 2, ns means no significant difference.

(a)

Query	Start-End	Peptide	M (calc)	M (expt)	m/z	Error	RT (min)	Area R-D	Missed	PTM
25	320-342	R.IGMEVTPSGTWLTYTAAIKLDDK.D	2510.8644	2509.9231	836.3452	-0.9413	43.55	4.66E+06	1	-
97	320-338	R.IGMEVTPSGTWLTYTAAIK.L	2039.3523	2038.8121	679.6631	-0.5402	13.44	2.59E+10	0	-

(b)

Query	Start-End	Peptide	M (calc)	M (expt)	m/z	Error	RT (min)	Area R-D	Missed	PTM
13	294-319	R.QGTDYKHWPQIAQFAPSASAFFGMSR.I	2945.1134	2944.3234	981.3242	-0.7900	53.55	5.62E+06	1	Oxidation (M)
78	348-361	K.DQVILLNKHIDAYK.T	1670.9211	1670.2148	835.3415	-0.7063	41.44	4.34E+06	1	Deamidation (NQ)
91	348-355	K.DQVILLNK.H	943.1455	943.6811	472.3413	0.5356	36.99	3.86E+10	0	Deamidation (NQ)
871	294-299	R.QGTDYK.H	710.7344	710.7441	355.7771	0.0097	2.32	7.31E+09	0	-

(c)

1 MSDNGPQNQR NAPRITFGGP SDSTGSNQNG ERSGARSKQR RPQGLPNNTA SWFTALTQHG
61 KEDLKFPRGQ GVPINTNSSP DDQIGYYRRA TRRIRGGDGK MKDLSPRWYF YYLGTGPEAG
121 LPYGANKDGI IWVATEGALN TPKDHIGTRN PANNAAIVLQ LPQGTTLPKG FYAEGSRGGS
181 QASSRSSSRS RNSSRNSTPG SSRGTSPARM AGNGGDAALA LLLLDRLNQL ESKMSGKGQQ
241 QQGQTVTKKS AAEASKKPRQ KRTATKAYNV TQAFGRRGPE QTQGNFGDQE LIRQGTDYKH
301 WPQIAQFAPS ASAFFGMSRI GMEVTPSGTW LTYTAAIKLD DKDPNFKDQV ILLNKHIDAY
361 KTFPPTEPKK DKKKKADETQ ALPQRQKKQQ TVTLLPAADL DDFSKQLQQS MSSADSTQA

(d)

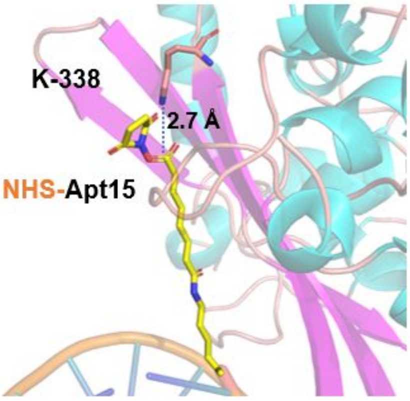

(e)

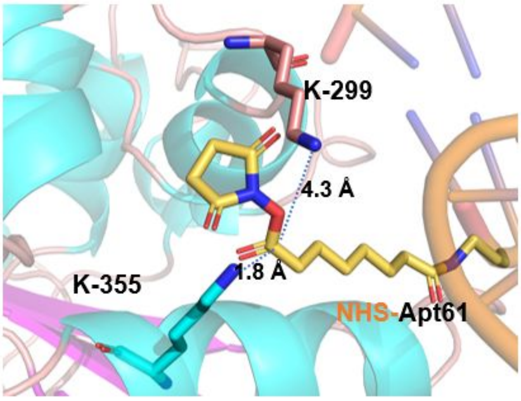

Figure S11. Analysis of crosslinking sites of NP and CNApts by LC-MS/MS experiment and molecular simulation. Analysis of crosslinking sites of NP and CNApts by LC-MS/MS experiment and molecular simulation. (a) LC-MS/MS results for extracted band of NHS-Apt15 crosslinking with NP. (b) LC-MS/MS results for extracted band of NHS-Apt61 crosslinking with NP. (c) Complete sequence and crosslinked amino acids (marked in red) of NP. (d-e) Molecular docking simulation of (d) NHS-Apt15 and (e) NHS-Apt61 binding with NP.

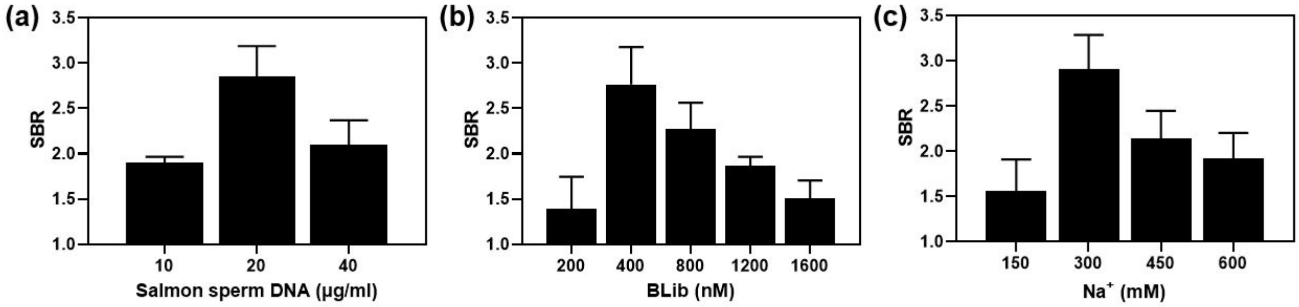

Figure S12. Optimization of concentrations of (a) electronegative salmon sperm DNA, (b) BLib DNA and (c) Na^+ of detection buffer to enhance specific binding ability in NApt-based ELISA assay. Herein SBR was defined as the ratio between sample signal (1 ng/mL NP) and background signal (0 ng/mL NP).

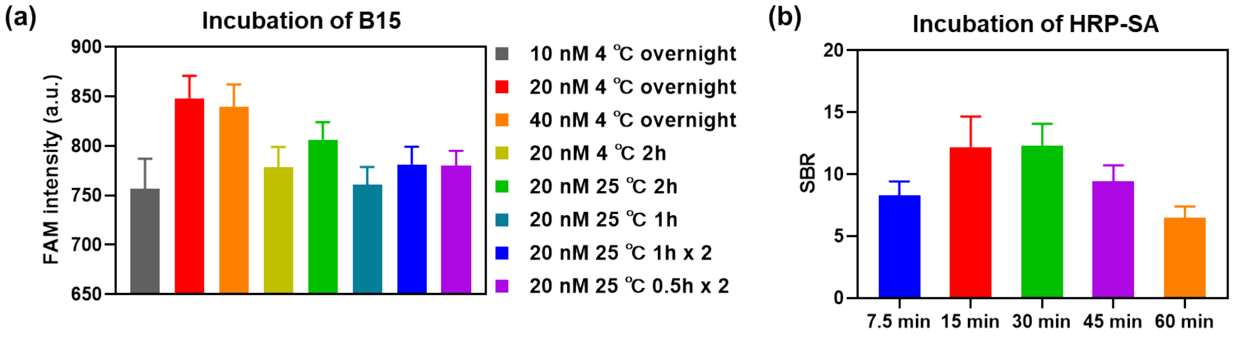

Figure S13. Optimization of the capture conditions and detection conditions in NApt-based ELISA assay. (a) The optimization of incubation method of capture aptamer (B15). Optimal one was 100 μL B15 at the concentration of 20 nM for preincubation at 4 °C overnight. (b) Optimization of incubation time of HRP-SA binding to B61. Herein SBR was defined as the ratio between sample signal (1 ng/mL NP) and background signal (0 ng/mL NP).

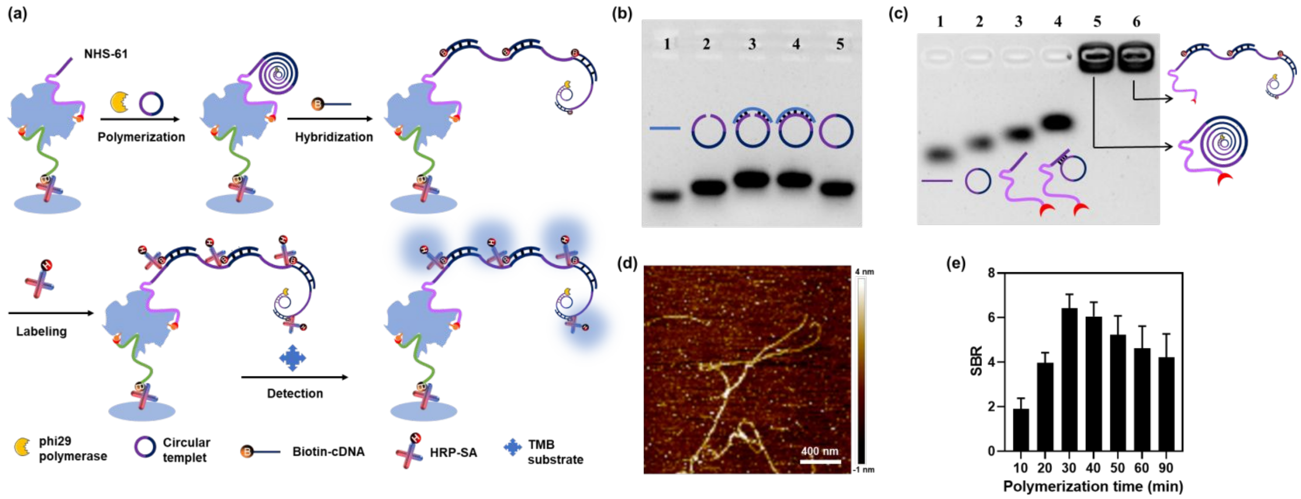

Figure S14. Design and characterizations of aptamer-based RCA. (a) Schematic illustration of covalent aptamer-based RCA ELISA. (b) Agarose gel electrophoresis analysis for preparation of circular template from padlock probe. Lane 1: ligation template (LT), lane 2: padlock probe (PP), lane 3: LT + PP, lane 4: lane 3 samples were treated with T4 ligase, lane 5: lane 4 samples were treated with Exo I and Exo III to obtain circular template (CT). (c) Agarose gel electrophoresis analysis of RCA reaction. Lane 1: cDNA; lane 2: CT; lane 3: NHS-61; lane 4: NHS-61+ CT; lane 5: lane 4+ phi29 DNA polymerase; lane 6: lane 5+ cDNA. (d) Atomic force microscope (AFM) imaging of RCA product. (e) Optimization of the amplification condition (polymerization time of phi29 DNA polymerase) by testing SBR of 1 pg/mL NP. Herein SBR was defined as the ratio between sample signal (1 pg/mL NP) and background signal (0 pg/ml NP). 30 min was the optimal condition.

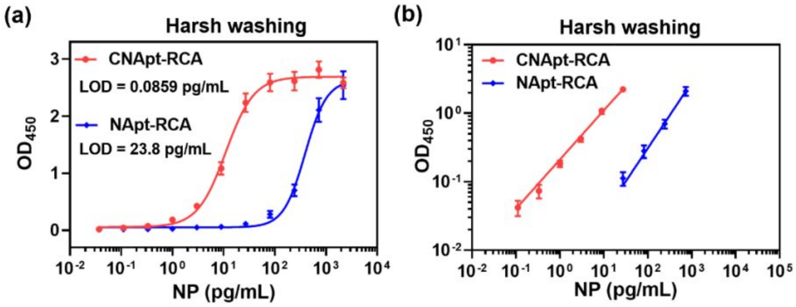

Figure S15. RCA technique-based sensitivity test and linear range analysis of NP dissolving in 10% FBS under harsh washing. (a) Sensitivity results under harsh washing. (b) Linear range results under harsh washing.

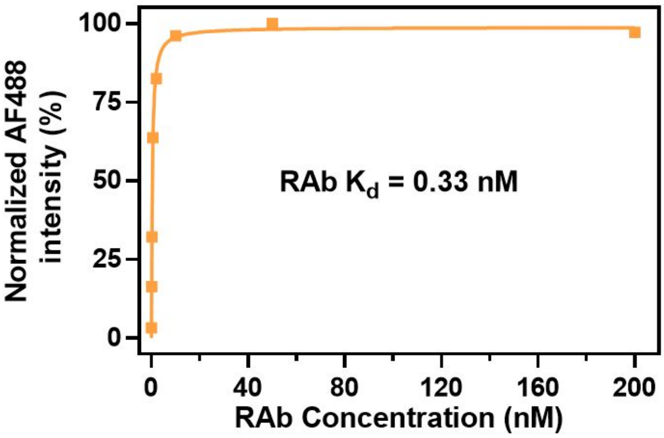

Figure S16. Binding affinity analysis of RAb against RBD protein at room temperature (RT).

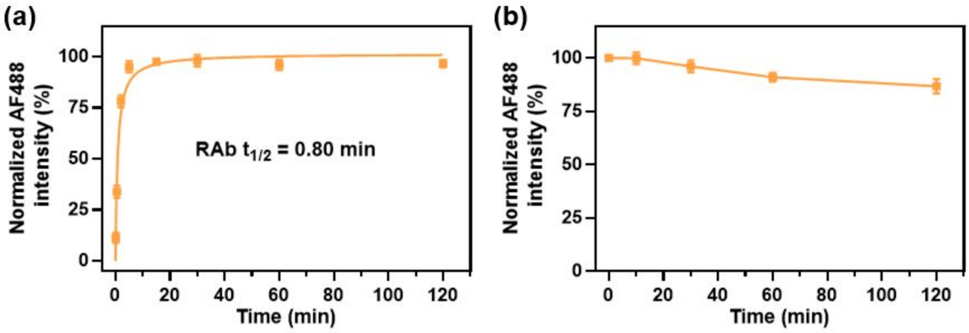

Figure S17. Binding kinetics and detachment situation analysis of RBD antibody (RAb) by flow cytometry tests. (a) Binding kinetics analysis of 2 nM RAb. All data are mean ± s.d., n = 2. (b) Detachment situation of 2 nM RAb from RBD bound complex after incubation in 10% FBS at 37 °C and multiple centrifugations. All data are mean ± s.d., n = 2.

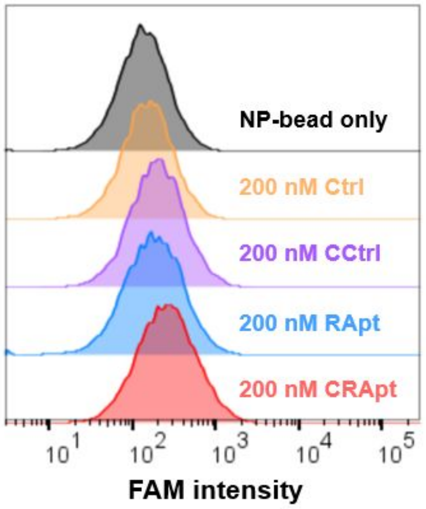

Figure S18. Binding events of NP-coated nickel microbead in flow cytometry assay.

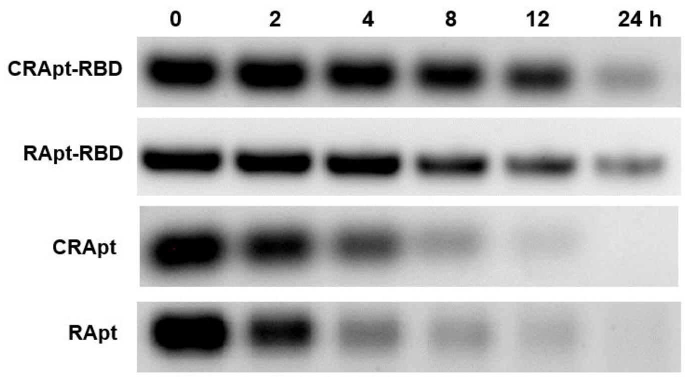

Figure S19. Stability test of aptamers and aptamer-RBD complexes in 10% FBS at 37 °C by AGE experiment.

(a)

Query	Start-End	Peptide	M (calc)	M (expt)	m/z	Error	RT (min)	Area R-D	Missed	PTM
59	409-424	R.QIAPGQTGKIADYNYK.L	1766.9521	1767.8999	883.9577	0.9478	23.59	6.64E+05	1	Deamidation (NQ)
217	409-417	R.QIAPGQTGK.I	899.0023	898.4872	450.2511	-0.5151	1.10	8.37E+10	0	-

(b)

1 MFVFLVLLPL VSSQCVNLTT RTQLPPAYTN SFTRGVYYPD KVFRSSVLHS TQDLFLPFFS
61 NVTWFHAIHV SGTNGTKRFD NPVLPFNDGV YFASTEKSNI IRGWIFGTTL DSKTQSLLIV
121 NNATNVVIKV CEFQFCNDPF LGVYYHKNNK SWMESEFRVY SSANNCTFEY VSQPFLMDLE
181 GKQGNFKNLR EFVFKNIDGY FKIYSKHTPI NLVRDLPQGF SALEPLVDLP IGINITRFQT
241 LLALHRSYLT PGDSSSGWTA GAAAYYVGYL QPRTFLLKYN ENGTITDAVD CALDPLSETK
301 CTLKSFTVEK GIYQTSNFRV QPTESIVRFP NITNLCPFGE VFNATRFASV YAWNRKRISN
361 CVADYSVLYN SASFSTFKCY GVSPTKLNDL CFTNVYADSF VIRGDEVRQI APGQTG**K**IAD
421 YNYKLPDDFT GCVIAWNSNN LDSKVGGNYN YLYRLFRKSN LKPFERDIST EIYQAGSTPC
481 NGVEGFNCYF PLQSYGFQPT NGVGYQPYRV VVLSFELLHA PATVCGPKKS TNLVKNKCVN
541 FNFNGLTGTG VLTESNKKFL PFQQFGRDIA DTTDAVRDPQ TLEILDITPC SFGGVSVITP
601 GTNTSNQVAV LYQDVNCTEV PVAIHADQLT PTWRVYSTGS NVFQTRAGCL IGAEHVNNSY
661 ECDIPIGAGI CASYQTQTNS PRRARSVASQ SIIAYTMSLG AENSVAYSNN SIAIPTNFTI
721 SVTTEILPVS MTKTSVDCTM YICGDSTECS NLLLQYGSFC TQLNRALTGI AVEQDKNTQE
781 VFAQVKQIYK TPPIKDFGGF NFSQILPDPS KPSKRSFIED LLFNKVTLAD AGFIKQYGDC
841 LGDIAARDLI CAQKFNGLTV LPPLLTDEMI AQYTSALLAG TITSGWTFGA GAALQIPFAM
901 QMAYRFNGIG VTQNVLYENQ KLIANQFNSA IGKIQDSLSS TASALGKLQD VVNQNAQALN
961 TLVKQLSSNF GAISSVLNDI LSRLDKVEAE VQIDRLITGR LQSLQTYVTQ QLIRAAEIRA
1021 SANLAATKMS ECVLGQSKRV DFCGKGYHLM SFPQSAPHGV VFLHVTYVPA QEKNFTTAPA
1081 ICHDGKAHFP REGVFVSNGT HWFVTQRNFY EPQIITTDNT FVSGNCDVVI GIVNNTVYDP
1141 LQPELDSFKE ELDKYFKNHT SPDVDLGDIS GINASVVNIQ KEIDRLNEVA KNLNESLIDL
1201 QELGKYEQYI KWPWYIWLGF IAGLIAIVMV TIMLCCMTSC CSCLKGCCSC GSCCKFDEDD
1261 SEPVLKGVKL HYT

(c)

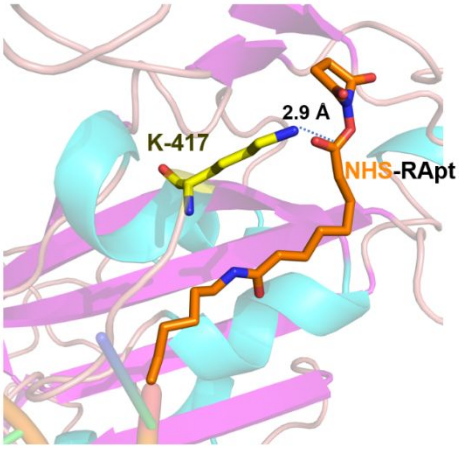

Figure S20. Analysis of crosslinking sites of CRApt against RBD protein by LC-MS/MS and molecular simulation. (a) LC-MS/MS results for extracted band of CRApt crosslinking with RBD. (b) Complete sequence and crosslinked amino acid (marked in red) of RBD. (c) Molecular docking simulation of CRApt binding with RBD.

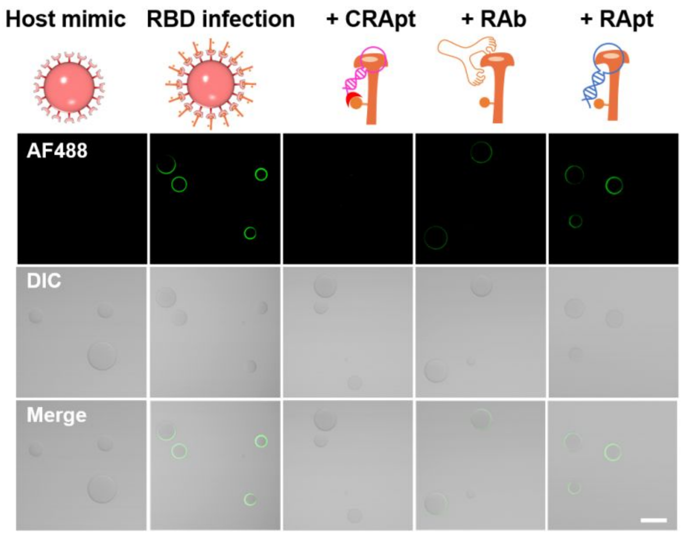

Figure S21. Confocal images of RBD neutralizers-mediated blocking effect. ACE2-coated nickel microbead was considered as host mimic. RBD protein was labeled by AF488 fluorescence. Scale bar, 50 μm.

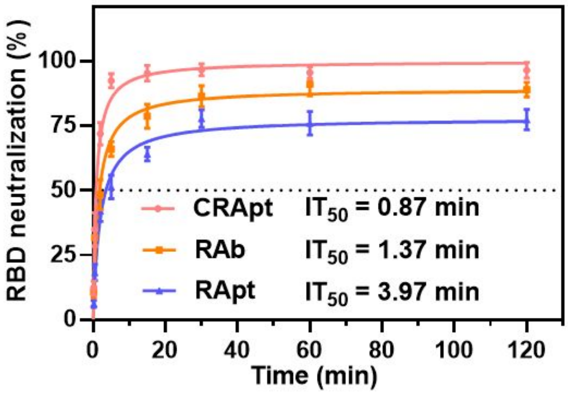

Figure S22. Flow cytometry analysis of half full inhibitory time (IT_{50}) of 200 nM CRApt, RAb and RApt. IT_{50} was defined as required time on 50% inhibitory rate.

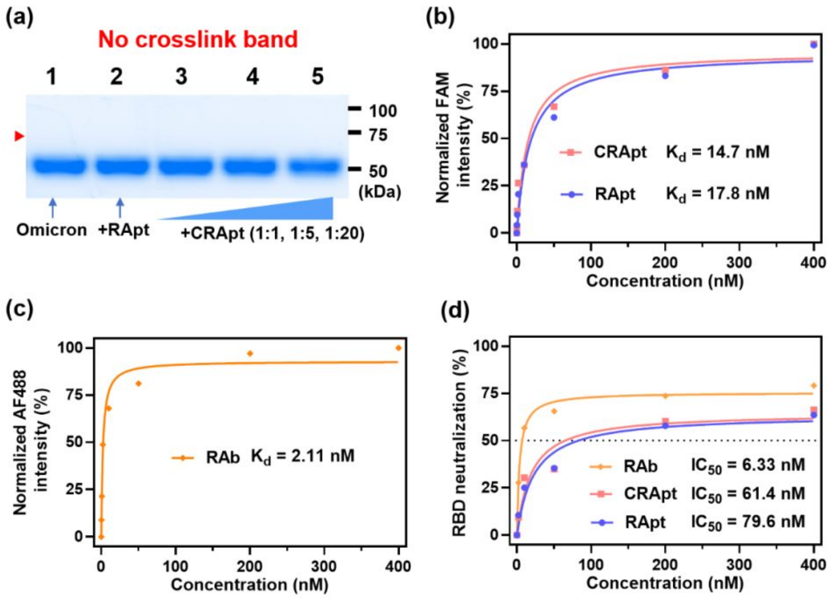

Figure S23. Binding ability and neutralization efficiency of CRApt against Omicron variant RBD protein. (a) Reducing SDS-PAGE analysis of crosslink efficiency after 400 nM Omicron variant RBD protein reacted with different concentrations of CNApt for 4 h (protein stained by Coomassie Blue). Lane 1: NP; lane 2: NP+ non-covalent NApt; lanes 3-5: NP+ 1, 5 and 10 equivalents of CNApt. (b) Binding affinity analysis of FAM-labeled CRApt and FAM-labeled RApt against Omicron variant RBD protein. (c) Binding affinity analysis of AF488-labeled RAb against Omicron variant RBD protein. (d) Neutralization analysis of different concentrations of RBD probe against Omicron variant RBD protein. All data are mean ± s.d., n = 3.

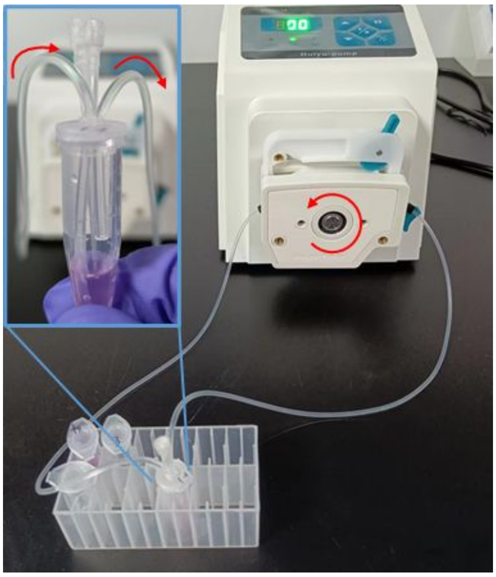

Figure S24. Constructing flowing circulation device by mimicking blood vessel. From the pipette tip, neutralizers, host mimic and RBD could be manually and alternatively added into the circulation system while the mixtures could be also pipetted out at different timepoints to measure the corresponding results.

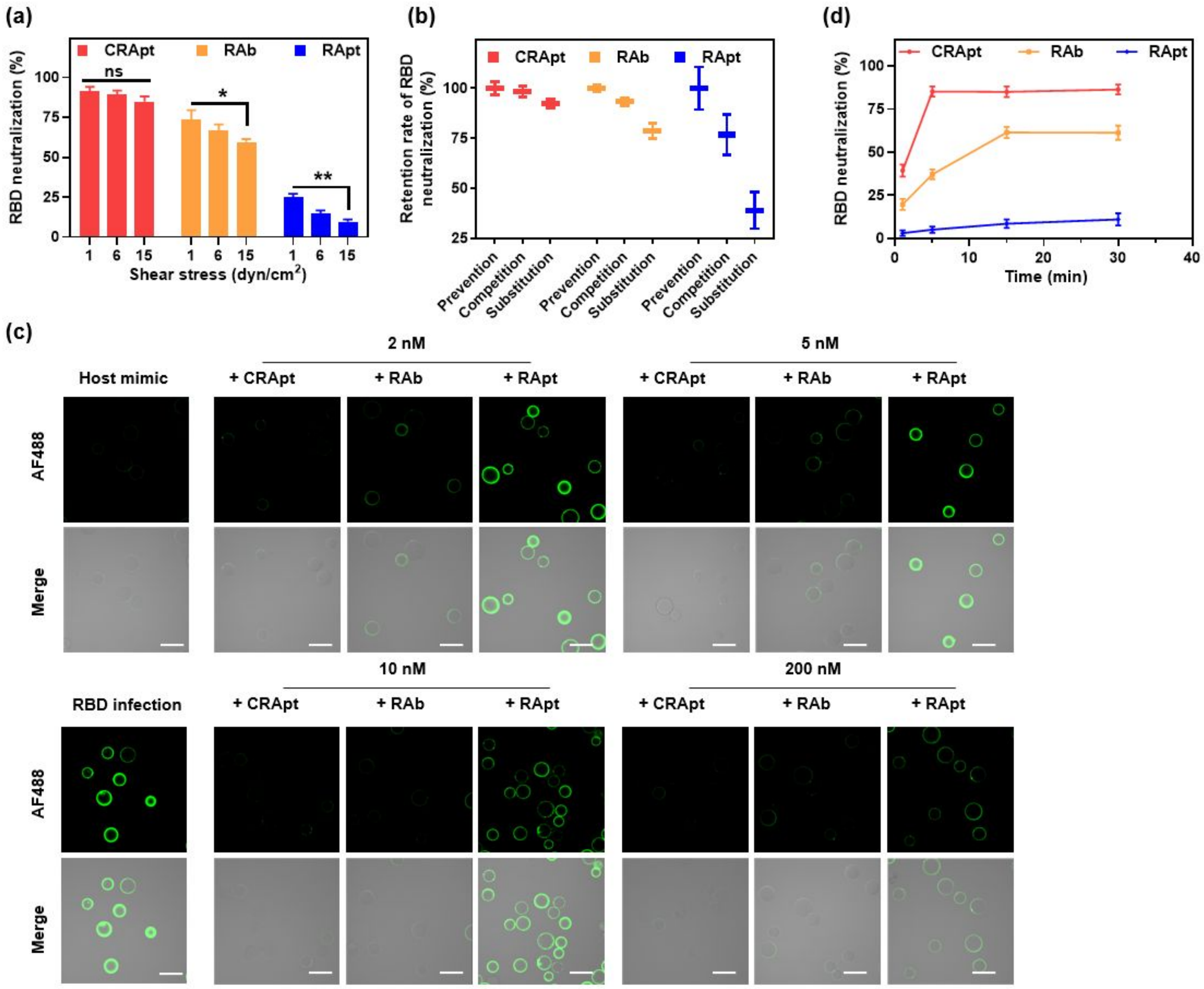

Figure S25. Neutralizing measurements under flowing circulation system. (a) RBD neutralizing analysis under different shear stress. (b) Retention rate analysis of RBD neutralization under different neutralizing modules. (c) Representative confocal imaging results of substitution-typed RBD neutralization analysis of different concentrations of RBD probes under the circulation system. (d) Neutralization speed analysis of different neutralizers under substitution module (therapy model).. All data are mean ± s.d., n = 2 in all statistical analysis. Statistical significance: $^{**}P < 0.01$, $^{*}P < 0.05$, ns means no significant difference.

3. Supporting Tables

Table S1. DNA sequences used in this work.

Name	DNA sequence (5'-3')	Modification or notes	Main applications
NApt (Apt61)[1]	GCT GGA TGT TGA CCT TTA CAG ATC GGA TTC TGT GGG GCG TTA AAC TGA CAC ATC CAG C	5' DBCO, or 5' NH_2, or (5' DBCO, 3' FAM), or (5' NH_2, 3' FAM)	For synthesizing CNApt
RApt	ATT ACC GAT GGC TTG TTT GTA ATG TAG GGT TCC GTC GGA T	5' DBCO, or 5' NH_2, or (5' DBCO, 3' FAM), or (5' NH_2, 3' FAM)	For synthesizing CRApt
B15	GCT GGA TGT TCA TGC TGG CAA AAT TCC TTA GGG GCA CCG TTA CTT TGA CAC ATC CAG CTT TTT TTT-Biotin	3' Biotin (or plus 5' FAM)	Selection of aptamer pairs, and optimization of incubation method of capture aptamer
B48	GCT GGA TGT CGC TTA CGA CAA TAT TCC TTA GGG GCA CCG CTA CAT TGA CAC ATC CAG CTT TTT TTT-Biotin	3' Biotin (or plus 5' FAM)	
B58	GCTGGATGTCACCGGATTGTCGGACATCGGATTGTCTGAGTCATATGACACATCCAGCtttttttt-Biotin	3' Biotin (or plus 5' FAM)	
B61	GCTGGATGTTGACCTTTACAGATCGGATTCTGTGGGGCGTTAAACTGACACATCCAGCtttttttt-Biotin	3' Biotin (or plus 5' FAM)	
BLib	NNNNNNNNNNNNNNNNNNN-Biotin	3' Biotin	Serve as binding buffer
NH_2-B15	NH_2-GCT GGA TGT TCA TGC TGG CAA AAT TCC TTA GGG GCA CCG TTA CTT TGA CAC ATC CAG Ctttttttt-Biotin	5' NH_2, 3' Biotin, for synthesizing NHS-B15	Gel electrophoresis analysis and ELISA assays
NH_2-B61	NH_2-GCTGGATGTTGACCTTTACAGATCGGATTCTGTGGGGCGTTAAACTGACACATCCAGCtttttttt-Biotin	5' NH_2, 3' Biotin, for synthesizing NHS-B61	
NH_2-BCtrl	NH_2-NNNNNNNNNNNNNNNNNNNNNNNNNNNNNNNNNNNNNNNNNNNNNNNNNNNNNNNNNNNNNNNNNNNNN-Biotin	5' NH_2, 3' Biotin, for for synthesizing NHS-BCtrl	
NH_2-Apt15	NH_2-GCTGGATGTTCATGCTGGCAAAATTCCTTAGGGGCACCGTTACTTTGACACATCCAGC-FAM	5' NH_2, 3' FAM, for synthesizing FAM-labeled NHS-Apt15	
NH_2-Apt61	NH_2-GCTGGATGTTGACCTTTACAGATCGGATTCTGTGGGGCGTTAAACTGACACATCCAGC-FAM	5' NH_2, 3' FAM, for synthesizing FAM-labeled NHS-Apt61	
NH_2-Ctrl	NH_2-NNNNNNNNNNNNNNNNNNNNNNNNNNNNNNNNNNNNNNNNNNNNNNNNNNNNNNNNNNNNNNNNNNNNN-FAM	5' NH_2, 3' FAM, for synthesizing FAM-labeled NHS-Ctrl	
Ligation probe	CTGTCCGTGCAAGGCCTGCC		RCA analysis and covalent aptamer-based ELISA
Padlock probe	p-GCACGGACAGttttCGTATCGTATGTTCGttttGGCAGGCCTT	5' Phosphate group	
Biotin-cDNA	Biotin-ttttttttCGTATCGTATGTTCG	5' Biotin	
NH_2-61	NH_2-GCTGGATGTTGACCTTTACAGATCGGATTCTGTGGGGCGTTAAACTGACACATCCAGCttttttttCTGTCCGTGCAAGGCCTGCC	5' NH_2 for synthesizing NHS-61	

Notes:

1. NApt was herein defined as Apt61 unless specially declared.
2. N represents arbitrary base, and library is a random ssDNA.
3. The underlined letters denote corresponding aptamer sequences.
4. NHS-Ctrl was also denoted to be CCtrl (covalent control DNA).

Table S2. Mass spectra of synthesized NHS-labeled aptamers.

Name	Calculated mass (Da)	Observed mass (Da)
SF-NApt	18613.2	18611.3
NHS-NApt	18335.8	18334.1
Acr-NApt	18136.8	18134.1
FAM-labeled SF-NApt	19182.7	19178.4
FAM-labeled NHS-NApt (NHS-Apt61)	18905.3	18902.7
FAM-labeled Acr-NApt	18706.3	18704.1
SF-RApt	13088.7	13091.1
NHS-RApt	12811.3	12813.8
Acr-RApt	12612.3	12610.1
FAM-labeled SF-RApt	13658.2	13656.9
FAM-labeled NHS-RApt	13380.8	13378.4
FAM-labeled Acr-RApt	13181.8	13184.2
NHS-B15	21068.8	21065.3
NHS-B61	21148.8	21144.6
FAM-labeled NHS-Apt15	18825.3	18828.1
NHS-61	26901.3	26903.4

Table S3. The comparison of CNApt ELISA and classic NAb ELISA under harsh washing.

Name	LOD (pg/mL)	Linear range (pg/mL)	Assay time (h)	Reagent cost per well ($)
CNApt ELISA	8.70	from 9.00 to 2187	2.5	0.14
NAb ELISA	70.0	from 81.0 to 2187	2.5	1.01

4. References

(1) Zhang, L., Fang, X., Liu, X., Ou, H., Zhang, H., Wang, J., Li, Q., Cheng, H., Zhang, W., Luo, Z. Discovery of Sandwich Type COVID-19 Nucleocapsid Protein DNA Aptamers. *Chem. Commun.* **2020,** *56*, 10235-10238.

(2) Cui, C., Zhang, H., Wang, R., Cansiz, S., Pan, X., Wan, S., Hou, W., Li, L., Chen, M., Liu, Y., Chen, X., Liu, Q., Tan, W. Recognition-Then-Reaction Enables Site-Selective Bioconjugation to Proteins on Live-Cell Surfaces. *Angew. Chem. Int. Ed.* **2017,** *56*, 11954-11957.
